# How to build a cold chain supply chain system for fresh agricultural products through blockchain technology—A study of tripartite evolutionary game theory based on prospect theory

**DOI:** 10.1371/journal.pone.0294520

**Published:** 2023-11-29

**Authors:** Yanhu Bai, Hansheng Wu, Minmin Huang, Jianli Luo, Zhuodong Yang

**Affiliations:** School of Business, Wenzhou University, Wenzhou, 325035, China; National Textile University, PAKISTAN

## Abstract

The global cold chain logistics market is witnessing a significant upswing, driven by the rising demand for perishable food products and increasing shipment volumes worldwide. Technological advancements are leading to a more intelligent and digitally enabled cold chain logistics system. However, the high loss rate of fresh agricultural products in China poses a significant threat to the country’s food security. Therefore, it is imperative to explore innovative solutions, such as blockchain, to address the challenges of traditional cold chain logistics. In this paper, inspired by the prospect theory and evolutionary game theory, we propose an evolutionary game model to analyze the behavioral strategies of the tripartite of n-level cold chain participants, consumers, and government. Using MATLAB software, the numerical simulation of the game path of this tripartite theory is conducted, and the influence of variable parameters on the evolutionary process and outcomes of the system is analyzed. The results the following: (1) The development of an effective cold chain supply chain system can be divided into three stages, and blockchain technology plays a pivotal role in creating a seamless cold chain environment. The cost of blockchain adoption, government rewards, as well as penalties can significantly influence the behavioral choices of the three stakeholders. (2) The behavior of individual cold chain participants has a strong negative externality, which can impact the behavior of others. We also find that the larger the scale of the cold chain, the lower the probability of default by the participants. (3) The government’s adoption of blockchain technology and the implementation of effective incentive policies can foster the successful development of the cold chain blockchain infrastructure. Our research contributes to the theoretical understanding of cold chain logistics decision making and policy creation for the tripartite stakeholders, including cold chain participants, consumers, and government. Our findings can serve as a valuable reference for scientific decision making and policy formulation to encourage the development of a robust cold chain supply chain system.

## 1. Introduction

Due to the perishable nature [1] and difficulty in storage [2] of fresh agricultural products, cold chain logistics is required [3] for their picking, packaging, storage, transportation, and distribution [4]. The cold chain is a vital supply chain system that includes upstream agricultural product suppliers and downstream agricultural product retailers [5]. However, information asymmetry and other issues in cold chain logistics lead to significant losses of agricultural products, making it imperative to develop and improve the cold chain logistics system to reduce these losses [6] and promote farmers’ income. According to Statista, the global cold chain logistics market was worth USD248.38 billion in 2020 and is projected to reach USD 410.79 billion by 2028. The total market size of China’s cold chain logistics industry was USD54.26 billion in 2020 and was expected to increase to USD 65.56 billion in 2022. Despite the significant advancements made in cold chain logistics monitoring technology, there remain issues in the data traceability of the fresh produce supply chain [7] that create a high probability of causing a "broken chain". Individual link participants in the cold chain logistics process may shut down refrigeration equipment or engage in other violations, which are difficult to identify through traditional monitoring means. Additionally, mismatches between supply and demand can cause significant waste of fresh produce [8]. Approximately one-third of the world’s food is wasted due to inefficient supply chains [9]. China has a relatively high rate of agricultural product loss during transit, with vegetables and fruits losing as much as 20% to 30% of their weight and meat losing as little as 12%. The loss of vegetables and fruits in China’s cold chain logistics process can exceed 100 billion yuan annually, resulting in significant losses for logistics enterprises as well as food waste. In the context of technological progress, cold chain logistics is moving towards more intelligent digitalization [10]. Therefore, the cold chain logistics industry urgently needs to establish a cold chain supply chain system (CCSCS) with traceable information using effective digital technology [11].

The Chinese government attaches great importance to the development of cold chain logistics. In April 2020, to ensure the construction of cold chain facilities for agricultural products storage and preservation, the Ministry of Agriculture and Rural Affairs issued the "Implementation Opinions of the Ministry of Agriculture and Rural Affairs on Accelerating the Construction of Cold Chain Facilities for Agricultural Products Storage and Preservation". In December 2021, the General Office of the State Council issued the "Fourteenth Five-Year Plan for the Development of Cold Chain Logistics", which sought to ensure the rapid construction of a modern cold chain logistics system by pressing the "fast-forward button" on the high-quality development of China’s fresh agricultural cold chain logistics. The government has promulgated relevant policies to strengthen regulation, management, and incentives; however, this has been challenging due to the backwardness of cold chain infrastructure, high logistics costs [12], extended supply chain of fresh agricultural products, lack of a perfect cold chain logistics information platform [13], information asymmetry between upstream and downstream enterprises [14], and frequent chain breakage [15]. There have also been policies implemented that focus on the role of consumers in the cold chain [16].

In the current context of imperfect cold chain technology, opaque information and complex relationship among all relevant stakeholders, many scholars have conducted research on cold chain logistics. And the research method of evolutionary game is heavily applied to the research in the field of cold chain logistics. Currently, cold chain logistics research is focusing its efforts on three key areas. First is, the application of digital technologies such as blockchain in cold chain logistics. Traditional cold chain logistics has problems such as centralized data storage, low data reliability, and easy data tampering, which leads to the inability to guarantee consumer rights [17]. Real-time information and the interconnectivity of elements, supplemented with novel technologies, will revolutionize and improve the way food logistics is carried out [18]. Blockchain technology uploads the data of each node to be stored in the server, and any individual block retains the data of the whole blockchain intact. The generated data cannot be modified, falsified, or deleted, ensuring high trustworthiness [4]. Trollman et al. [19] examined how blockchain technology can be used to support the ecological embedding of the coffee supply chain. Kim et al. [20] proposed using service level agreements (SLAs) to produce performance evaluation metrics for contracts to prevent risk factors by using blockchain technology to measure and share information in real time. Hollands et al. [21] explored the significance of blockchain technology for the growth of the food industry and the limitations that it currently faces; this method significantly reduced the time to generate blocks and was suitable for platforms that collect and serve real-time information and apply it in cold chain logistics. The second key area is the analysis of factors affecting whether and how to better facilitate small downstream information interchange among cold chain participants. The cold chain participants form a node in the cold chain logistics, and whether the cold chain participants fulfill their commitment to open cold storage facilities will determine whether the cold chain will have a "broken chain" or not. Shen et al. [22] used evolutionary game theory to study the effect of government incentives on information exchange and sharing among cold chain participating firms. Zhang [23] simulated two different types of fresh products based on a pricing model, compared two strategies of cooperative and non-cooperative games, and explored the profit changes of fresh e-commerce and cold chain companies in different price ranges and using selected pricing strategies. Liu et al. [24] established an evolutionary game model of cold chain logistics outsourcing for fresh produce enterprises with operational risks and found that factors such as initial state, cost of default and loss of unfulfilled contracts can have an impact on cold chain logistics enterprises. The third key factor is the influence of consumer aspects on the cold chain. Consumers’ demand for diversity, nutrition, and taste of food stimulates the development of the cold chain. In addition, the quality of the product that consumers finally buy will directly affect consumers’ evaluation of that cold chain and determine whether the cold chain is effective or not. Wang et al. [12] discussed the role of consumers’ food safety awareness and suggested that the government should encourage consumers to raise their food safety awareness to stimulate cold chain investment and thus ensure food safety. Rong et al. [25] showed that as China’s income and education levels increase, consumers will become more accepting of paying the cost of cold chain logistics and will pay relatively higher premiums. Jo et al. [26] analyzed 12 meta-values of consumers and proposed a food cold chain management strategy to meet consumer perceptions.

In summary, the existing studies have explored in some depth the feasibility of the application of blockchain technology in cold chain logistics and which factors of relevant stakeholders will affect the construction of CCSCS, providing a solid theoretical foundation for this paper on the research of constructing CCSCS under blockchain technology. Nonetheless, the following represent limitations of the current literature. Firstly, the majority of cold chain studies focus on the connection between one variable (such as consumers or businesses) and the cold chain. Few studies combine data from many participants into a single analytical framework. Second, there are few studies on the direct interaction between consumers and cold chain participants, particularly the government’s influence on consumers or cold chain participants in terms of policies. Third, few papers consider the mutual impact caused by the negative externalities of each node in the cold chain on other nodes and the deviation of the perceived benefits of the game subjects in practice.

To fill the relevant research gap mentioned above, this paper introduces prospect theory to the current Chinese cold chain situation and uses it to build a tripartite evolutionary model of n-level cold chain participants, consumers, and government. In particular, we address the following issues: (1) In a replicative dynamic system including cold chain participants, consumers, and the government, what is the evolutionary stabilization strategy (ESS), and what are the important elements affecting the ESS? (2) How do the system’s internal variables influence the system overall? (3) What steps should the government take to build a strong CCSCS?

The following are the paper’s most significant contributions: (1) We propose a new framework for collaborative development that brings together stakeholders from the cold chain, including n-level cold chain participants, consumers and government. (2) We study the conditions for achieving the optimal steady state of the cold chain supply chain by observing the effect of their interaction mechanisms on the system. (3) We simulate the system to see how different parameters would affect it and use these to deduce policy changes that would help establish a functional cold chain supply system at the national level. (4) In order to further the science of decision-making for governments, cold chain participants, and consumers, we add prospect theory and introduce the perceived biases brought about by psychological considerations.

The remainder of this paper is structured as follows: In Section 2: Model building on theoretical basis, we lay out the study issue and build an evolutionary model with n-level cold chain actors, n-level consumers, and n-level policymakers as the three axes. Section 3: Model analysis: Expected payoff and replicator dynamics equation of each participant analyzes the model’s possible payoff scenarios with respect to the system of replicator dynamics equations. The evolutionary equilibrium and the system stability point of the three parties are analyzed in Section 4: Stability analysis of evolutionary games, which also depicts the modeling method of the evolutionary game. In Section 5: Simulation Analyses of the Evolutionary Game, we simulate the effects of changing various parameters of the system and analyze the steps the government should take in response. Section 6: Discussion presents the discussion of simulation results. In Section 7: Conclusions, we review the paper’s key findings, offer policy recommendations that follow from those findings, and critically examine the paper’s shortcomings and the outlook for future investigation.

## 2. Model building on theoretical basis

### 2.1 Evolutionary game theory

Evolutionary game theory [27] explains the phenomenon of mutual learning, competition, and adaptation in the evolution of organisms. In contrast to traditional game theory, which emphasizes static equilibrium, it focuses on the analysis of the dynamic process of system equilibrium, including the path to reaching equilibrium and the specific developments and changes in the system. In contrast to traditional game theory, which emphasizes perfect rationality, in evolutionary game theory, participants are assumed to be finitely rational and eventually seek evolutionary stability strategies through continuous trial and error, imitation, and learning [28].

Due to the non-transparent and non-sharing information of the cold chain supply chain and the information asymmetry of the upstream and downstream cold chain participants, the behavior of a single cold chain participant may cause massive externalities, which eventually affect the whole cold chain. Although the Chinese government has prioritized the construction of cold chains and has formulated a series of policies to promote the establishment of CCSCS, the behavioral choices of cold chain participants are also influenced by the preferences of consumers and other cold chain participants. The behavioral strategies of governments, cold chain participants, and consumers are unpredictable, as they do not have complete control over the information in their environment. They can only make the best judgment under a process of continuous "learning". Therefore, evolutionary game theory is widely used in cold chain-related research. Ma [29] established a dynamic game model of a cold chain formed by a milk manufacturer and two downstream oligopolistic supermarkets. Zhao [30] analyzed the impact of multiple factors on electric refrigerated truck(ERT) upgrading and penetration strategies by building an evolutionary game model. The stakeholders involved in establishing the CCSCS under blockchain technology are cold chain participants, consumers, and the government. The game is constantly repeated among the three parties. Given that evolutionary game theory can analyze the strategy and choices of each participant under conditions of cooperation and conflict, the theory can be used to analyze the behavior and game process of cold chain participants, consumers, and the government.

Matlab is a powerful numerical computing and scientific programming software that offers a wide range of features and tools for data processing, analysis, and visualization, as well as solving various engineering and scientific problems. It is widely used by engineers, scientists, and researchers in the fields of science and engineering [31].

In this study, we utilized Matlab for simulation analysis based on the replicator dynamics system to provide theoretical foundations for the model. This method has been widely recognized and applied by numerous scholars in various fields. Evolutionary game theory, based on the assumption of bounded rationality, can effectively reflect the strategy selection of participants in real-life situations. Over many years of research, the use of Matlab software has become increasingly sophisticated, and a well-developed and reasonable analysis methodology has been established, capable of addressing various analytical problems. In various research studies, many scholars have applied Matlab for analysis. For instance, Liu et al. [32] used MATLAB software to numerically simulate the paths of tripartite game. Luo et al. [33] utilized MATLAB R2018b to primarily showcase the strategic selection trends of logistics companies in various scenarios, including "government failure," as well as the mutual impact of strategy selection between the government and environmental non-governmental organizations.

### 2.2 Prospect theory

Traditional games theorys mostly uses the concept of expected returns to analyze the strategy choices of game subjects; however, in practice, there are deviations from the perceived returns of game subjects in practice. Prospect theory, on the other hand, can analyze decision makers’ judgments in light of the uncertainty of payoffs. Prospect theory is a descriptive technique that originated in psychology and has become an alternative theory to utility theory and other classical approaches to decision making under risk and uncertainty [34]. Unlike the "rational man" hypothesis long used in economics, prospect theory explains the influence of irrational factors on human judgment and decision making according to people’s psychological and behavioral characteristics [35]. Prospect theory has been applied to economics, evolutionary biology, and political science [36]. A large number of scholars have analyzed the decision making behavior of game subjects in the game process through prospect theory. For example, Li [37] combined prospect theory to construct an evolutionary game model of vaccination and studied the role of perception in promoting vaccination.

Government’s policy has an impact on the cold chain construction environment. However, the government’s interests may deviate from those of other participants, so the gains and losses brought to the cold chain participants and consumers are uncertain, and their behavioral gains and losses are subject to perception bias. When faced with risk, individuals compare actual utility with that of a reference point and make decisions based on the results. Due to limited rationality, it is difficult for cold chain participants and consumers to find the best strategy in the game. They can only determine the best strategy gradually through continuous learning.

Both prospect theory and evolutionary game theory are based on the premise of finite rationality. The combination of these two theories can reflect the decision-making psychology of government, cold chain participants, and consumers when facing uncertain profit and loss and can dynamically describe their strategic choice process. In addition, we use simulation to study the influence of different factors on their strategic choices. Because of this, it is appropriate to introduce prospect theory to construct a game model of CCSCS evolution and adopt subjective probability weight function and value function to measure perceived benefits, which can more realistically reflect the actual perceptions of game subjects.

### 2.3 Problem description

On the whole, the development of China’s cold chain logistics industry started late compared with that of developed countries, and there remains a great gap in terms of development. Although China’s cold chain logistics has made apparent progress in terms of industrial scale, development quality, innovation pace, and market competitiveness. However, development is hampered by various issues, such as a lack of standards in some areas of cold chain logistics, the low level of cold chain technology [38], outdated cold chain equipment [39], an unsound cold chain logistics system in large and medium-sized cities [40], the lack of an agricultural logistics information platform [13]. Thus, there is no way to guarantee a constant cold temperature along the whole supply chain of cold chain logistics. In addition, decentralized, individual farmer production is still the norm in the agriculture sector. When it comes to moving and selling agricultural goods, especially perishables, supply and marketing cooperatives have little more than a symbolic presence.

Cold chain participants: Cold chain participants at all levels are the essential components of the cold chain, and different participants correspond to various nodes in the cold chain. Food producers and retailers rarely collect systematic data [41], and the information is challenging to trace under the traditional cold chain regulation model. There are high speculative gains for cold chain participants who do not keep their contracts. The negative externality of cold chain participants’ default will affect other cold chain participants’ willingness to keep their contracts, which will negatively affect the whole cold chain system.

Government: The government is the decision maker of the CCSCS. A perfect information traceability system ensures that when problems occur in the cold chain, the problematic links can be pinpointed and dealt with. Therefore, the government’s adoption of blockchain policy will also provide oversight for cold chain participants, prompting them to keep their contracts. The construction of the cold chain blockchain system is influenced by multiple participants, and the behavioral choices of each party will have an impact on the whole system; how government formulates policy to guide this system will play a key role [22]. The reasonableness of the government’s reward and punishment system [30] will also affect the motivation of consumers to report and determine whether the cold chain participants are in breach of contract.

Consumers: Whether the cold chain participants fulfill their commitments will affect the quality of fresh produce as purchased by consumers; thus, consumer reporting can regulate the behavior of cold chain participants to a certain extent. Consumers’ willingness to report will be affected by consumers’ basic understanding of the cold chain system [42] and food safety awareness [43], as well as the soundness of the legal system.

### 2.4 Model assumptions

**Fig 1:**
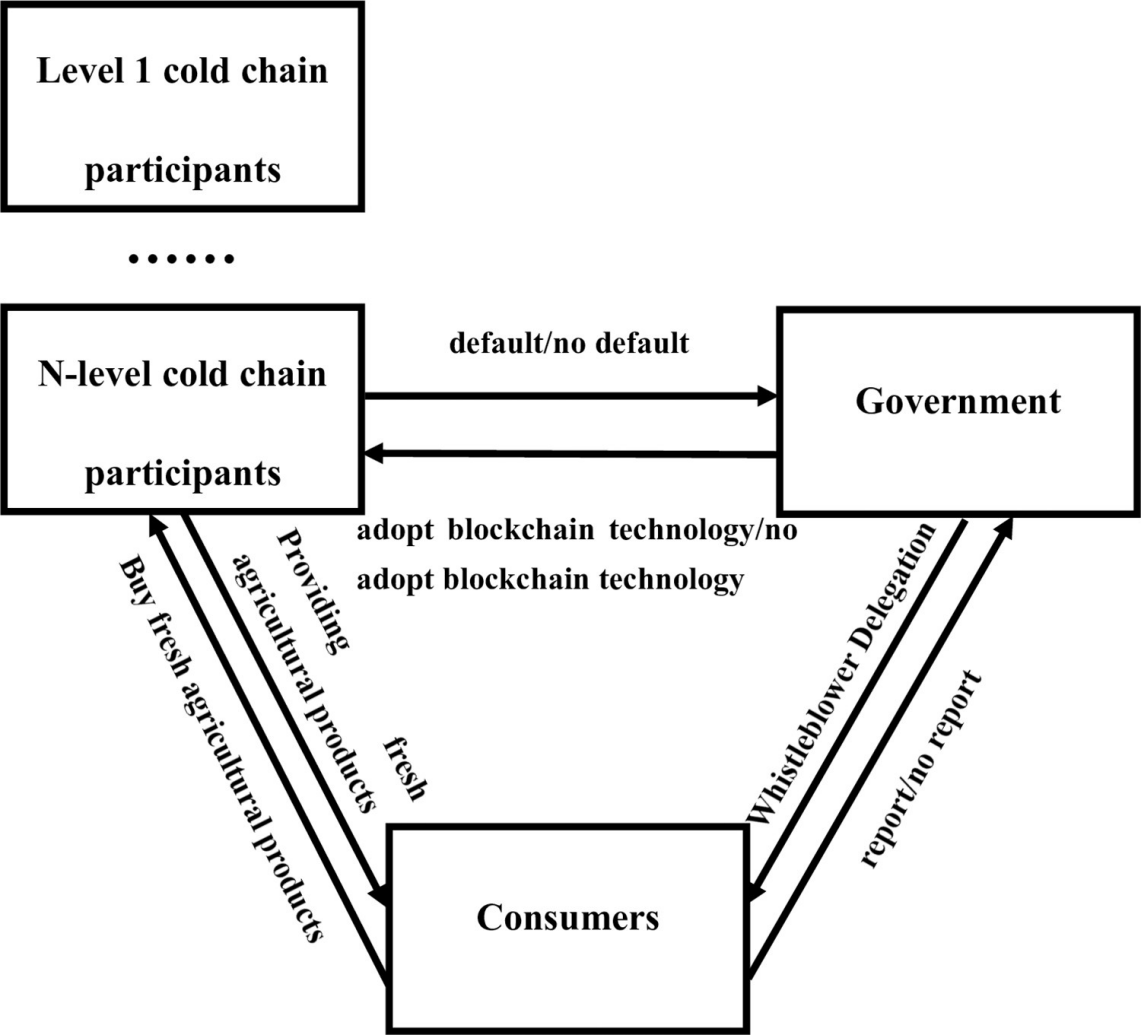
Logical diagram of the interrelationships between the entities in a tripartite evolutionary game.

The logical relationship between the tripartite evolutionary game subjects of building the CCSCS constructed in this paper is shown in Fig 1. The fresh produce supply chain is composed of various participants. This includes the production, processing, transportation, storage and sales of agricultural products, etc. Through the cold chain logistics, fresh produce will be provided to consumers, while consumers will have the right to choose to report problems in the fresh produce provided, and the government will accept the report entrusted by consumers and identify them, and the government can choose whether to use blockchain technology, which will have an impact on whether it can be successfully identified.

Assumption 1: The game participants are n-level cold chain participants (x), consumers (y), and government (z), and all three parties are finite rational.

Assumption 2: The strategy space of n-level cold chain participants is (default (x), no default (1-x)); the strategy space of fresh produce consumers is (report (y), no report (1-y)); the strategy space of government is (adopt blockchain technology (z), no adopt blockchain technology (1-z)).

Assumption 3: The three subjects make strategic choices based on their perceived benefits, and two models from prospect theory are cited as the basis for measuring perceived benefits. Prospect theory was jointly proposed by Tversky and Kahneman [44] to explain decision-making behavior in the case of limited rationality. The theory converts probabilities into weights based on expected returns using a subjective probability weight function and replaces the utility model with a value function. The expected value in the prospect theory is shown in Eq (1).

U=∑w(p)v(Δπ) (1)

where p represents the objective probability of event i, and w(p) means the decision weight of the decision maker’s subjective measure of event i, as a monotonically increasing function, as in Eq (2). Usually, one assigns a weight of 1 to very high probability events and a weight of 0 to shallow probability events, then one has w(1) = 1 and w(0) = 0. While medium and high probability events are easily underestimated, and low probability events are overestimated, one has w(p) < p when Pi is large and w(p) > p when Pi is tiny. V(Δπ) represents the subjective value perceived by the decision maker, as in Eq (3).


$$w(p)=\frac{p^{\gamma }}{({\left(p^{\gamma }+{\left(1-p\right)}^{\gamma }\right)}^{1/\gamma }}$$ (2)

$$V(\Delta \pi )=\{\begin{array}{l} \Delta \pi^{\alpha },\Delta \pi \geq 0\\ -\lambda {\left(-\Delta \pi \right)}^{\beta },\Delta \pi <0 \end{array}\}$$ (3)

The function of Eq (2) is an inverted "S" shape, and the smaller γ is, the more curved the function graph is. In Eq (3), the parameter Δπ represents the difference in returns before and after the game. The parameters α, β ∈ (0,1) represent the risk preference coefficients of decision-makers, and the larger the value, the higher the sensitivity to loss and gain. λ (λ ≥ 1) is the loss avoidance coefficient, and the larger the value, the higher the sensitivity to loss than gain.

Assumption 4: The participants in the cold chain constitute the entire chain and are present at every stage, making their number quite large, which makes them an ideal subject for replicating dynamic research. Many studies have used companies as subjects for replicating dynamic research in numerous literature. Shan et al. [45] establish a tripartite evolutionary game model of PV enterprises, poor households, and the government. The revenue that n-level cold chain participants can obtain by selling units of fresh agricultural products is Pr, and the total quantity of agricultural products transported in the cold chain is Qu. The probability of default of participants at the previous levels is α, the loss rate of fresh agricultural products caused by the default of the earlier participants is β, and the quantity of fresh agricultural products finally obtained by the nth-level participants is $Q_{x}=Q_{u}*{\left(1-\beta \right)}^{(n-1)*\alpha }$. The cost of turning on the cold storage equipment of the n-level cold chain participants is Co, and the default of the participants The existence of speculative gain is Sp. When the default of the participant is reported, a fine of Fc will be imposed on the defaulting cold chain participant, and the total number of penalties for the first n-1 level participants is $F_{x}=F_{c}*\alpha *(n-1)$. Meanwhile, if the government does not adopt blockchain technology will provide a favorable environment Gb to the cold chain participants.

Hypothesis 5: Consumers are the largest group of participants in the cold chain system, and their behavioral patterns are gradual and can have significant impacts on other participants. Many classic studies have used consumers as their research subjects. For example, Long et al. [46] build a tripartite game model involving government, consumers and enterprises. Each level of participant default will cause an inevitable loss $h_{o}^{i}$ to the fresh produce purchased by consumers, and the loss caused by the default of a participant in the cold chain transportation is $H_{l}=\sum_{i=1}^{n-1}h_{0}^{i}$, and the loss of the n-level is Hn. The perceived benefit of the fresh produce purchased by consumers is Pe. Consumers can choose to report the participants in the cold chain, and when a participant in the cold chain defaults, the government will reward the consumers who report successfully Sx. The government adopts blockchain technology will provide vital support to ensure the legitimate rights and interests of consumers Mc.

Assumption 6: As the implementer and regulator of policies, the government also adjusts its policies in response to changing situations. In many classic evolutionary game theory articles, the government is regarded as a participant. For example, Wang et al. [47] constructed a three-party evolutionary game model consisting of the government, recyclers, and consumers, while Sheng et al. [48] constructed a three-party evolutionary game model consisting of the central government, local governments, and enterprises in China. The government needs to pay a particular technical cost Ck to adopt blockchain technology, and when the government does not adopt blockchain technology but the consumer reports, it needs to invest the price of Cf to identify the probability of successful identification is λ. The revenue that the fresh produce will bring to the market for the government is P.

According to prospect theory, there is no deviation between actual utility and perceived value when the benefits or losses are certain; in contrast, there is only psychologically perceived utility when the decision maker is uncertain about the benefits and costs. The cost of adopting blockchain technology by the government, the cost of identifying the reports after receiving them, and the fines and rewards for other participants are interrelated and are deterministic expenditures. Meanwhile, the revenue from selling fresh produce and the cost of turning on the cold storage equipment of the n-level cold chain participants are also only interrelated and are deterministic expenses. Therefore, Pr, Co, Qu, Fc, Mg, Hn, Sx, Ck, Cf, P, α, β, and λ do not have perceptual bias, while Sp, Gb, Pe, and Mc have uncertainty and perceptual characteristics. This results in a specific combination of n-level cold chain participants, consumers, and government strategies, as well as the corresponding perceived benefits, as shown in Table 1. The specific parameters are described in Table 2.

**Table 1:** Three-party hybrid strategy game model for building CCSCS by blockchain technology.

Strategy Selection		consumers	government		
			adopt blockchain technology z	no adopt blockchain technology 1-z	
**n-level cold chain participants**	default x	report y	V(Sp)+Pr*Qx*(1-β)-Fc V(Pe)+Sx+V(Mc)-Hl-Hn P-Ck+Fc-Sx+Fx		V(Gb)+V(Sp)+Pr*Qx*(1-β)-Fc*λ V(Pe)+Sx*λ-Hl-Hn P-Cf+Fc*λ-Sx+Fx
		no report 1-y	V(Sp)+Pr*Qx*(1-β) V(Pe)+V(Mc)-Hl-Hn P-Ck-Fx		V(Gb)+V(Sp)+Pr*Qx*(1-β) V(Pe)-Hl-Hn P
	no default 1-x	report y	V(Sp)+Pr*Qx+Mg-Co V(Pe)+α*Sx+V(Mc)-Hl P-Ck+Fx-Mg		V(Sp)+Pr*Qx+Mg-Co V(Pe)+α*λ*Sx-Hl P-Cf+Fx-Mg
		no report 1-y	V(Sp)+Pr*Qx-Co V(Pe)+V(Mc)-Hl P-Ck		V(Sp)+Pr*Qx-Co V(Pe)-Hl P

**Table 2:** Evolutionary game model parameter variable.

Parameter	n-level cold chain participants
Sp	Speculative gains from defaults
Pr	Gain on sale of unit quantity of produce
α	Chance of possible default by a former participant
β	Chance of loss of fresh produce due to default by a former participant
Co	Costs required to turn on refrigeration equipment
Qu	Amount of produce transported
Gb	Non-adoption of blockchain policy to provide an enabling environment for default
Fc	Penalty for participant default
Mg	Government incentives for cold chain participants not to default
Qx	Quantity of produce for n levels of participants
Fx	Total number of participant fines at the first n-1 levels
	**Consumers**
$h_{o}^{i}$	Certain losses due to participant default at each level
Hl	Losses due to default by a participant in cold chain transport
Hn	Losses due to default by participants in the first n levels of cold chain transport
Pe	Perceived benefits of purchasing fresh produce
λ	Probability of successful identification and reporting by the government
Sx	Proceeds from successful reporting
Mc	Strong support to ensure consumer rights after the adoption of blockchain policy
	**Government**
Ck	Technical cost of adopting blockchain technology
Cf	Cost of detection without blockchain technology
P	Benefits of fresh produce reaching the market

## 3. Model analysis: Expected payoff and replicator dynamics equation of each participant

Based on the payoff matrix of the evolutionary game model, we can obtain the expected and average payoffs of the three subjects, n-level cold chain participants, consumers and government, which we denote by Eij and $\bar{E_{i}}$respectively:

E11=(1‐z)*V(Gb)+V(Sp)+(λ‐1)*z‐λ*y*Fc+Pr*Qx*(1‐β) (4)

E12=(‐1+z)*(‐1+y)*V(Sp)+(y*(1‐z)+z)*V(Sp)+Mg*y+Pr*Qx‐Co (5)

E21=x*z*(V(Pe)+Sx+V(Mc)‐Hl‐Hn)+x*(1‐z)*(V(Pe)+SX*λ‐Hl‐Hn)+(1‐x)*z*(V(Pe)+α*Sx+V(Mc)‐Hl)+(1‐x)*(1‐z)*(V(Pe)+α*λ*Sx‐Hl) (6)

E22=x*z*(V(Pe)+V(Mc)‐Hl‐Hn)+x*(1‐z)*(V(Pe)‐Hl‐Hn)+(1‐x)*z*(V(Pe)+V(Mc)‐Hl)+(1‐x)*(1‐z)*(V(Pe)‐Hl) (7)

E31=x*y*(P‐Ck+Fc‐Sx+Fx)+x*(1‐y)*(P‐Ck‐Fx)+(1‐x)*y*(P‐Ck+Fx‐Mg)+(1‐x)*(1‐y)*(P‐Ck) (8)

E32=x*y*(P‐Cf+Fc*λ‐Sx+Fx)+x*(1‐y)*(P)+(1‐x)*y*(P‐Cf+Fx‐Mg)+(1‐x)*(1‐y)*(P) (9)

E1=x*E11+(1‐x)*E12 (10)

E2=y*E21+(1‐y)*E22 (11)

E3=z*E31+(1‐z)*E32 (12)

Since there is asymmetry in the information available to n-level cold chain actors, consumers, and the government, all three parties will make their own decisions based on constant trial and error learning and historical experience to infer the strategies of the other parties. A replication process that is itself dynamic, with x, y, and z all making changes to their own strategies, as defined by evolutionary game theory.

Replicator dynamics equations are mathematical models used to describe the changes in strategy distribution during the evolution of a population [49]. They serve as a vital tool in evolutionary game theory [50], widely applied in studying collective behavior in social, economic, and biological systems. In replicator dynamics, each strategy or behavior is assigned a proportion or frequency within the population, representing its relative prevalence. These proportions change over time, reflecting the competition and selection among different strategies during the evolutionary process.

Following is the formula for the replication dynamic equation of n-level cold chain actors, consumers, and governments:

$$F(x)=\frac{\mathrm{dx}}{\mathrm{dt}}=x*(E11‐E1)=x*(x‐1)*(Mg*y‐V(Gb)‐Co+z*V(Gb)+\beta *Pr*Qx+\lambda *Fc*y+Fc*y*z‐\lambda *Fc*y*z)$$ (13)

$$F(y)=\frac{\mathrm{dy}}{\mathrm{dt}}=y*(E21‐E2)=‐Sx*y*(y‐1)*(\alpha +x‐\alpha *x)*(\lambda +z‐\lambda *z)$$ (14)

$$F(z)=\frac{\mathrm{dz}}{\mathrm{dt}}=z*(E31‐E3)=z*(z‐1)*(Ck‐Cf*y+Fx*x‐Fc*x*y‐Fx*x*y+\lambda *Fc*x*y)$$ (15)

## 4. Stability analysis of evolutionary games

### 4.1 Stable strategy analysis for each participant

Stability strategy analysis is a method used to evaluate the stability and evolutionary outcomes of strategies in game theory [51]. It is an important tool in the study of games and decision problems, helping us understand the dynamic changes of strategies and the behavioral evolution of game participants. The core idea of stability strategy analysis is to assess the interactions and outcomes among different strategies, determining which strategies can maintain their relative advantages and stability in long-term evolution. It takes into account the interactions and feedback effects among game participants, as well as their process of selecting and adjusting strategies.

When (x, y, z) no longer varies over time, the decision made by each participant is the best possible strategy, as predicted by the differential equation stability principle when the replica dynamic equation is 0 and its first derivative is smaller than 0. As a result, the following is a stable study of the tactics employed by the n-tiered cold chain’s actors, customers, and governments:

For the n-level cold chain participants, the following conclusions can be drawn from Eq (10):

1. When $0<y<\frac{\left(\beta {*P}_{r}{*Q}_{x}{+z*V(G}_{b}{)‐C}_{o}{‐V(G}_{b})\right)}{\left(\lambda {*F}_{c}*z‐\lambda {*F}_{c}{‐F}_{c}{*z‐M}_{g}\right)}$, then ${\frac{dF(x)}{dx}|}_{x=1}<0$, ${\frac{dF(x)}{dx}|}_{x=0}>0$, it can be inferred that x = 1 is the evolutionary stable point of the n-level cold chain participants. It shows that the n-level cold chain participants’ strategy changes from no default to default, and finally stabilized by choosing to default.
2. When $y=\frac{\left(\beta {*P}_{r}{*Q}_{x}{+z*V(G}_{b}{)‐C}_{o}{‐V(G}_{b})\right)}{\left(\lambda {*F}_{c}*z‐\lambda {*F}_{c}{‐F}_{c}{*z‐M}_{g}\right)}$, then *F*(*x*) = 0. It shows that the n-level cold chain participants choose to default and do not have the same benefit. All x are in an evolutionarily stable state, and the policy choice does not change with time.
3. When $\frac{\left(\beta {*P}_{r}{*Q}_{x}{+z*V(G}_{b}{)‐C}_{o}{‐V(G}_{b})\right)}{\left(\lambda {*F}_{c}*z‐\lambda {*F}_{c}{‐F}_{c}{*z‐M}_{g}\right)}<y<1$, then ${\frac{dF(x)}{dx}|}_{x=0}<0$, ${\frac{dF(x)}{dx}|}_{x=1}>0$, it can be inferred that x = 0 is the evolutionary stable point of the n-level cold chain participants. It shows that the n-level cold chain participants’ strategy changes from default to no default, and finally stabilized by choosing no default.

For the consumers, the following conclusions can be drawn from Eq (11):

1. When $0<z<\frac{\lambda }{\lambda ‐1}$, then ${\frac{dF(y)}{dy}|}_{y=1}>0$, ${\frac{dF(y)}{dy}|}_{y=0}<0$, it can be inferred that y = 0 is the evolutionary stable point of the consumers. It shows that the consumers’ strategy changes from report to no report, and finally stabilized by choosing no report.
2. When $z=\frac{\lambda }{\lambda ‐1}$, then *F*(y) = 0. It shows that the consumers choose to report and do not have the same benefit. All y are in an evolutionarily stable state, and the policy choice does not change with time.
3. When $\frac{\lambda }{\lambda ‐1}<z<1$, then ${\frac{dF(y)}{dy}|}_{y=0}>0$, ${\frac{dF(y)}{dy}|}_{y=1}<0$, it can be inferred that y = 1 is the evolutionary stable point of the consumers. It shows that the consumers’ strategy changes from no report to report, and finally stabilized by choosing to report.

For the government, the following conclusions can be drawn from Eq (12):

1. When $0<x<\frac{{(C}_{f}{*y‐C}_{k})}{\left(\lambda {*F}_{c}{*y‐F}_{c}{*y‐F}_{x}{*y+F}_{x}\right)}$, then ${\frac{dF(z)}{dz}|}_{z=1}<0$, ${\frac{dF(z)}{dz}|}_{z=0}>0$, it can be inferred that z = 1 is the evolutionary stable point of the government. It shows that the government’s strategy changed from not adopting blockchain technology to adopting blockchain technology and finally stabilized by choosing to adopt blockchain technology.
2. When $x=\frac{{(C}_{f}{*y‐C}_{k})}{\left(\lambda {*F}_{c}{*y‐F}_{c}{*y‐F}_{x}{*y+F}_{x}\right)}$, then *F*(z) = 0. It shows that the government chooses to adopt blockchain technology and does not have the same benefit. All z are in an evolutionarily stable state, and the policy choice does not change with time.
3. When $\frac{{(C}_{f}{*y‐C}_{k})}{\left(\lambda {*F}_{c}{*y‐F}_{c}{*y‐F}_{x}{*y+F}_{x}\right)}<x<1$, then ${\frac{dF(z)}{dz}|}_{z=0}<0$, ${\frac{dF(z)}{dz}|}_{z=1}>0$, it can be inferred that z = 0 is the evolutionary stable point of the government. It shows that the government’s strategy changes from adopting blockchain technology to not adopting blockchain technology, and finally stabilized by choosing to not adopt blockchain technology.

### 4.2 System stability analysis

When F (x) = F (y) = F (z) = 0, we can obtain the local equilibrium points as E1 (0,0,0), E2 (0, 0,1), E3 (0.1,0), E4 (1,0,0), E5 (0,1,1), E6 (1,0,1), E7 (1,1,0), and E8 (1,1,1). According to the method proposed by Friedman [52], the local stability of the equilibrium point can be analyzed by the Jacobian matrix of the system. According to the evolutionary game theory, the equilibrium point that satisfies all the eigenvalues of the Jacobian matrix are non-positive is the e evolutionary stabilization strategy (ESS) of the system. The specific analysis is as follows:

$$J=(\begin{array}{ccc} \frac{d(F(x))}{\mathrm{dx}} & \frac{d(F(x))}{\mathrm{dy}} & \frac{d(F(x))}{\mathrm{dz}}\\ \frac{d(F(y))}{\mathrm{dx}} & \frac{d(F(y))}{\mathrm{dy}} & \frac{d(F(y))}{\mathrm{dz}}\\ \frac{d(F(z))}{\mathrm{dx}} & \frac{d(F(z))}{\mathrm{dy}} & \frac{d(F(z))}{\mathrm{dz}} \end{array})$$ (16)

$$\left\{ \begin{array}{l} \frac{d(F(x))}{\mathrm{dx}}=x*(M_{g}*y‐V(G_{b})‐C_{o}+z*V(G_{b})+\beta *P_{r}*Q_{x}+\lambda *F_{c}*y+F_{c}*y*z‐\lambda *F_{c}*y*z)+\left(x‐1\right)*(M_{g}*y‐V(G_{b})‐C_{o}+z*V(G_{b})+\beta *P_{x}*Q_{x}+\lambda *F_{c}*y+F_{c}*y*z‐\lambda *F_{c}*y*z)\\ \frac{d(F(x))}{\mathrm{dy}}=x*(x‐1)*(M_{g}+F_{c}*z+\lambda *F_{c}‐\lambda *F_{c}*z)\\ \frac{d(F(x))}{\mathrm{dz}}=x*(x‐1)*(V(G_{b})+F_{c}*y‐\lambda *F_{c}*y)\\ \frac{d(F(y))}{\mathrm{dx}}=S_{x}y*\left(\alpha ‐1\right)*\left(y‐1\right)*(\lambda +z‐\lambda *z)\\ \frac{d(F(y))}{\mathrm{dy}}=‐S_{x}*y*\left(\alpha +x‐\alpha *x\right)*(\lambda +z‐\lambda *z)‐S_{x}*\left(y‐1\right)*\left(\alpha +x‐\alpha *x\right)*(\lambda +z‐\lambda *z)\\ \frac{d(F(y))}{dz}=S_{x}*y*\left(\lambda ‐1\right)*\left(y‐1\right)*(\alpha +x‐\alpha *x)\\ \frac{d(F(z))}{\mathrm{dx}}=z*(z‐1)*(F_{x}‐F_{c}*y‐F_{x}*y+\lambda *F_{c}*y)\\ \frac{d(F(z))}{dy}=‐z*(z‐1)*(C_{f}+F_{c}*x+F_{x}*x‐\lambda *F_{c}*x)\\ \frac{d(F(z))}{\mathrm{dz}}=\left(z‐1)*(C_{k}‐C_{f}*y+F_{x}*x‐F_{c}*x*y‐F_{x}*x*y+\lambda *F_{c}*x*y)+z*(C_{k}‐C_{f}*y+F_{x}*x‐F_{c}*x*y‐F_{x}*x*y+\lambda *F_{c}*x*y\right) \end{array}\right.$$ (17)

The Jacobian matrix serves as a fundamental tool for assessing the stability of evolution [53]. At an equilibrium point, the eigenvalues of the Jacobian matrix, denoted as J, play a crucial role in determining its stability. Specifically, if all eigenvalues of J are less than 0, the equilibrium point represents an evolutionary stable strategy. Conversely, if all eigenvalues are greater than 0, the equilibrium point is deemed unstable. If one or two of the eigenvalues are greater than 0, it corresponds to a saddle point.

The above eight equilibrium points represent eight different scenarios. It is evident that E1 (0,0,0) E2 (0,0,1) E4 (1,0,0) E6 (1,0,1), and E8 (1,1,1) cannot be evolutionarily stable points. E3 (0,1,0), E5 (0,1,1), and E7 (1,1,0) are possible evolutionarily stable points, and these three points correspond to the cold chain. Although the stable strategy for consumers in all three situations is to report, simulation analysis reveals that the evolutionary paths of consumers in the three situations are not the same and that consumers have a constraining effect on the other two parties that cannot be ignored. The three development stages in the construction of the supply chain system are analyzed as follows. The eigenvalues of all equilibrium points are shown in Table 3.

**Table 3:** Stability of equilibrium points.

Equilibrium	Jacobian Eigenvalues
	λ1,λ2,λ3
E1(0,0,0)	Co + V(Gb) -β*Pr*Qx, α*λ*Sx, -Ck
E2(0,0,1)	Co -β*Pr*Qx, α*Sx, Ck
E3(0,1,0)	Co—Mg + V(Gb) -λ*Fc -β*Pr*Qx, -α*λ*Sx, Cf—Ck
E4(1,0,0)	β*Pr*Qx—V(Gb)—Co, λ*Sx,—Ck—Fx
E5(0,1,1)	Co—Fc—Mg -β*Pr*Qx, -α*Sx, Ck—Cf
E6(1,0,1)	β*Pr*Qx—Co, Sx, Ck + Fx
E7(1,1,0)	Mg—Co—V(Gb) +λ*Fc +β*Pr*Qx, -λ*Sx, Cf—Ck + Fc -λ*Fc
E8(1,1,1)	Fc—Co + Mg +β*Pr*Qx, -Sx, Ck—Cf—Fc +λ*Fc

Case 1: At this point the condition Mg—Co—V(Gb) + λ*Fc + β*Pr*Qx,—λ*Sx, Cf—Ck + Fc—λ*Fc is satisfied. (1, 1, 0) shows that in the case that the government does not use blockchain technology, the cold chain participant chooses to default, and the consumer chooses to report.

This case is equivalent to the early stage of the construction of the CCSCS, due to an underdeveloped industry culture and environment, the supply chain technology is not sufficiently mature, and the cost to the government in adopting blockchain technology is high. When the cost to the government in adopting blockchain technology is surpasses a certain threshold, Cf—Ck + Fc—λ*Fc < 0, the government will not adopt the blockchain policy. As the government does not adopt blockchain policy, it provides favorable conditions for cold chain participants to default; at this time, cold chain participants can achieve more considerable speculative gains with lower hypothetical costs, and the government’s insufficient rewards and punishments make the original gains of default higher than those of non-default. At this time, Mg—Co—V(Gb) + λ*Fc + β*Pr*Qx < 0, and cold chain participants will choose to default. Due to the lack of corresponding government measures, the probability of default of cold chain participants becomes higher and higher, and consumers will suffer losses as a result; thus, to protect their rights and interests, consumers will choose to report.

Case 2: At this point the condition Co—Fc—Mg—β*Pr*Qx, -α*Sx, Ck—Cf is satisfied. (0, 1, 1) shows that in the case where the government uses blockchain technology, the cold chain participant chooses not to breach the contract, and the consumer chooses to report.

This case is equivalent to the development period. With the gradual maturity of blockchain technology and the government’s awareness of the importance of strengthening the construction of the CCSCS, the government will begin to increase the supervision of the cold chain. When the cost of using blockchain technology is less than traditional regulations to identify cold chain problems, Ck—Cf < 0, and the government will start to adopt blockchain technology. At the same time, the government also starts strengthening the rewards and penalties for cold chain participants. When the bonuses and punishments reach a certain level, Co—Fc—Mg—β*Pr*Qx < 0, the cold chain participants will choose not to breach the contract. At this time, there remain some cold chain participants in breach of contract, and consumers will choose to report under the dual role of reward for successful reporting and protection of their rights and interests.

Case 3: At this point the condition Co—Mg + V(Gb) -λ*Fc -β*Pr*Qx, -α*λ*Sx, Cf—Ck is satisfied. (0, 1, 0) indicates that in the case that the government does not use blockchain technology, the cold chain participant chooses not to breach the contract, and the consumer chooses to report.

This case is equivalent to the mature stage of the development of the cold chain industry. blockchain technology is developed, and the government attaches great importance to it. The benefits of default of the cold chain participants will be significantly reduced, and they will start to consciously comply with the industry norms. At this time Co—Mg + V(Gb)—C*Fc—B*Pr*Qx <0, and the cold chain participants will choose not to default. Additionally, at this time, excessive government intervention will only increase the financial burden, and since Cf—Ck < 0, the government will gradually withdraw from the market and choose not to adopt blockchain technology. Consumers will still choose to report problems with the product due to the low cost of reporting and the reward for successful reporting.

**Fig 2:**
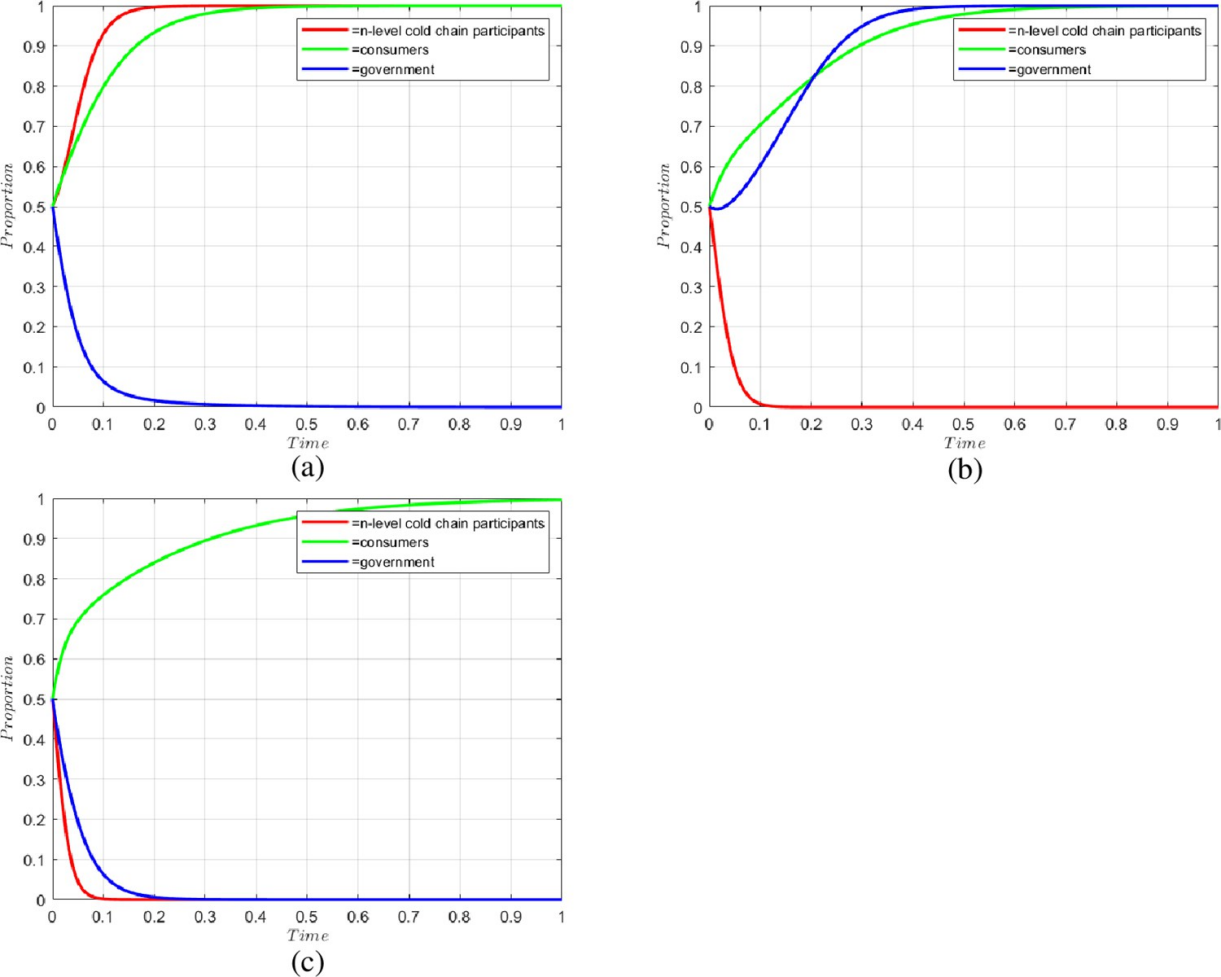
Changes in the willingness of system parties under different conditions: (a) Case 1; (b) Case 2; (c) Case 3.

The simulations of the three cases are shown in Fig 2.

## 5. Simulation Analyses of the Evolutionary Game

### 5.1 Initial variable

This section discusses the sensitivities of critical parameters in the system stability analysis, including the cost required to adopt blockchain technology, the amount of raw produce to be transported, and the chance of default for former cold chain participants. In modeling the sensitivity of the parameters, we hold the other parameters constant.

HNA Cold Chain is a company dedicated to third-party cold chain logistics for warehousing, transportation, and distribution services, as well as providing logistics solutions and other value-added logistics services to its customers. Since its establishment in 1997, based on the rapidly developing industry environment, the company has been improving its domestic and international network layout, focusing on becoming a comprehensive service provider, cold chain ecological operator, and investor in the global production, financing, and trust integration of the cold chain industry, such as Zhejiang’s Wenling watermelon, yellow cauliflower, and other special agricultural products sold to the whole country and the world through the cold chain. And in the future, it will further standardize the corporate governance structure and operation management, accelerate the construction and network layout of the national cold chain logistics center, while continuously extending to the business fields of cold chain finance, cold chain business, and cold chain technology, and transforming from a traditional logistics resource operator to a modern cold chain logistics resource integrator. This case is typical and replicable for the cold chain industry in China and worldwide. Therefore, we choose HNA Cold Chain Company as the case for calculating the initial values of variables.

In this paper, the initial values of the parameters are obtained through four channels. First, the relevant policies promulgated by the government. Opinions on the Implementation of Rewards for Reporting Cold Chain Logistics Epidemic" encourages the whole society to report the illegal acts of cold chain logistics, and once the reported clues are confirmed, the reward amount will be calculated according to 1% of the amount of administrative punishment, and the maximum reward can be 50,000 yuan, and we refer to get Sx = 20. The National Development and Reform Commission issued the "About the Construction of National Backbone Cold Chain Logistics Base in 2020 The Notice of Work" mentioned that in 2020, the Ministry of Agriculture and Rural Affairs, together with relevant departments, arranged 5 billion yuan of central financial funds to support 16 provinces, such as Hebei and Shanxi, to carry out the construction of storage and preservation cold chain facilities around fresh and live agricultural products such as fruits and vegetables, and we refer to get Mg = 20. Second, the classical literature and financial statements of enterprises. The data obtained by Liu [24] through the interview of "SF Express", we refer to get the following parameter Sp = 50. Through the evolutionary game model established by Song [54], which consists of government, telecommunication operators, and agricultural enterprises, we refer to get Ck = 30. Through the study of the supply chain logistics information collaboration strategy by Zhang [55], we refer to get Hn = 15. Third. Data from field research. We researched more than ten cold chain-related companies and one thousand consumers of fresh products in China and got α = 0.3, β = 0.2, λ = 0.4, and Pe = 100. Fourth, the opinions of experts in related fields. More than ten experts in related fields in Wenzhou combined the data obtained from the research with a large amount of related literature to estimate the impact of government policies on cold chain participants and consumers in terms of objective environment and subjective factors. For the sake of validity and accuracy of the data, we grouped these more than ten experts, and then performed several valuations and optimization processes to obtain the most reasonable data.

With the above four sources, we finally determined the initial data of each parameter and simplified the process, as shown in Table 4.

**Table 4:** Initial values of all variables.

Participants	Parameters	Variables	Value
n-level cold chain participants	Speculative gains from non-compliance	Sp [24]	50
	Gain from selling a unit quantity of produce	Pr [56]	3
	Chance of possible default by a former participant	α(Field research)	0.3
	Rate of loss of fresh produce due to default by a former participant	β(Field research)	0.2
	Costs r to turn on refrigerated equipment for transport	Co [56]	45
	Amount of produce transported	Qu [56]	100
	Non-adoption of blockchain policy to provide an enabling environment for default	Gb(Expert assessment)	20
	Penalty for participant default	Fc(Expert assessment)	20
	Incentives	Mg [57]	20
Consumers	The nth level of loss is	Hn [55]	15
	Perceived benefits of purchasing fresh produce	Pe(Field research)	100
	Probability of successful identification and reporting by the government	λ(Field research)	0.4
	Gains from successful reporting	Sx [58]	20
	Strong support to ensure consumer rights with the adoption of blockchain policies	Mc(Expert assessment)	30
Government	Technical cost of adopting blockchain technology	Ck [54]	30
	Cost of detection without blockchain technology	Cf(Expert assessment)	40
	Benefits of fresh produce reaching the market	P(Expert assessment)	100

### 5.2 Simulation of changes in different variables

#### 5.2.1 Impact of the cost required to adopt blockchain technology

**Fig 3:**
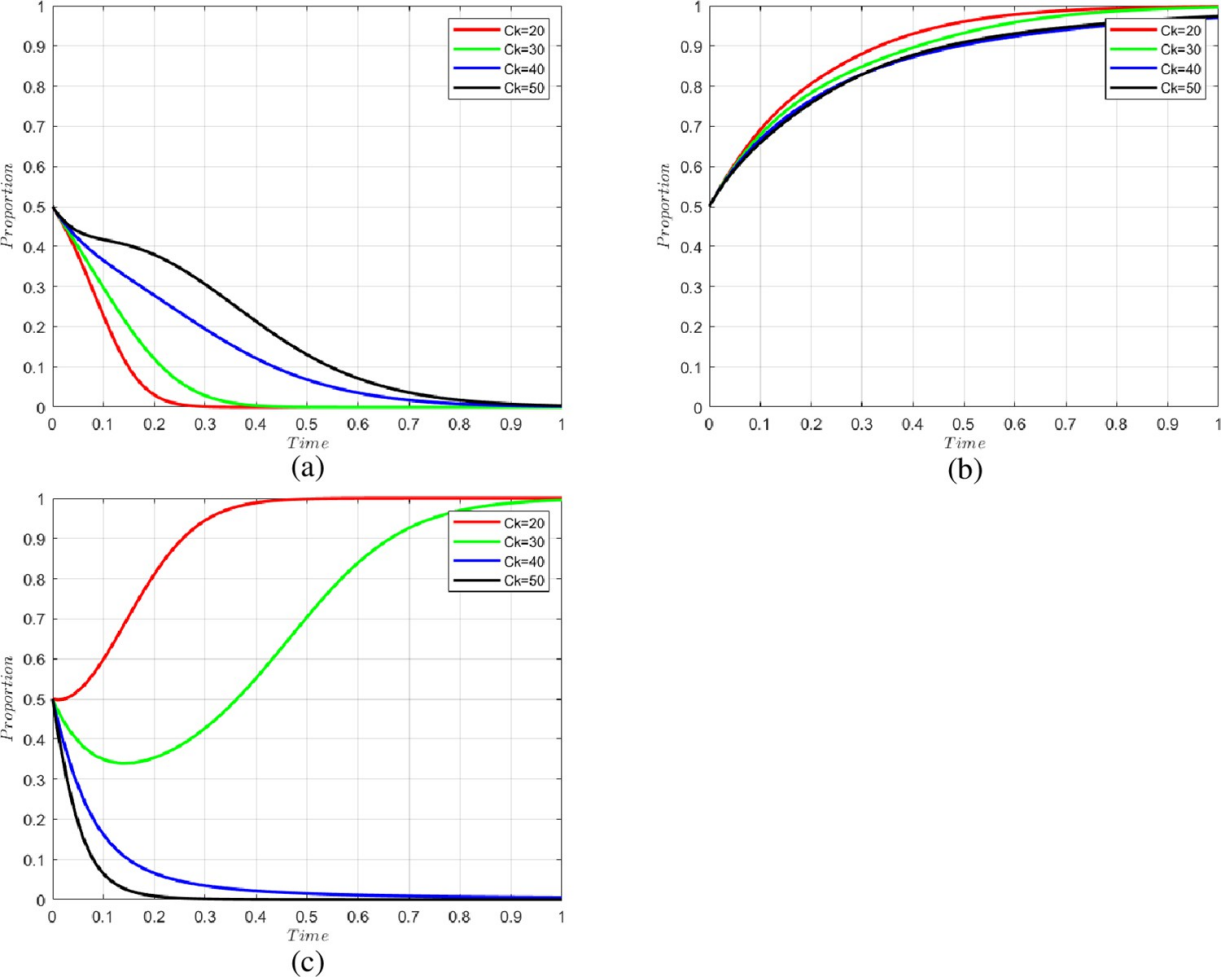
Change in the willingness of parties at different costs of adopting blockchain technology. (a) change in the willingness of n-level cold chain participants, (b) change in the willingness of consumers, and (c) change in the willingness of government.

We set the cost of adopting blockchain technology as Ck = 20, 30, 40, 50; the change of willingness of each party is shown in Fig 3. From the figure, we can see that the technical cost of blockchain technology adoption by the government will significantly affect the willingness of the government to adopt blockchain technology, and the willingness of the government to adopt blockchain technology will increase significantly as the cost of blockchain technology decreases. Blockchain technology can establish a perfect cold chain information tracing system, accurately supervise the CCSCS, and effectively identify problematic links. Therefore, the government’s willingness to adopt blockchain technology will also affect the probability of successful reporting by consumers, which in turn will affect consumers’ willingness to report. Therefore, vigorously developing blockchain technology and reducing the cost of using blockchain technology will have a substantial impact on building a CCSCS.

#### 5.2.2 The impact of transporting the quantity of fresh agricultural products

**Fig 4:**
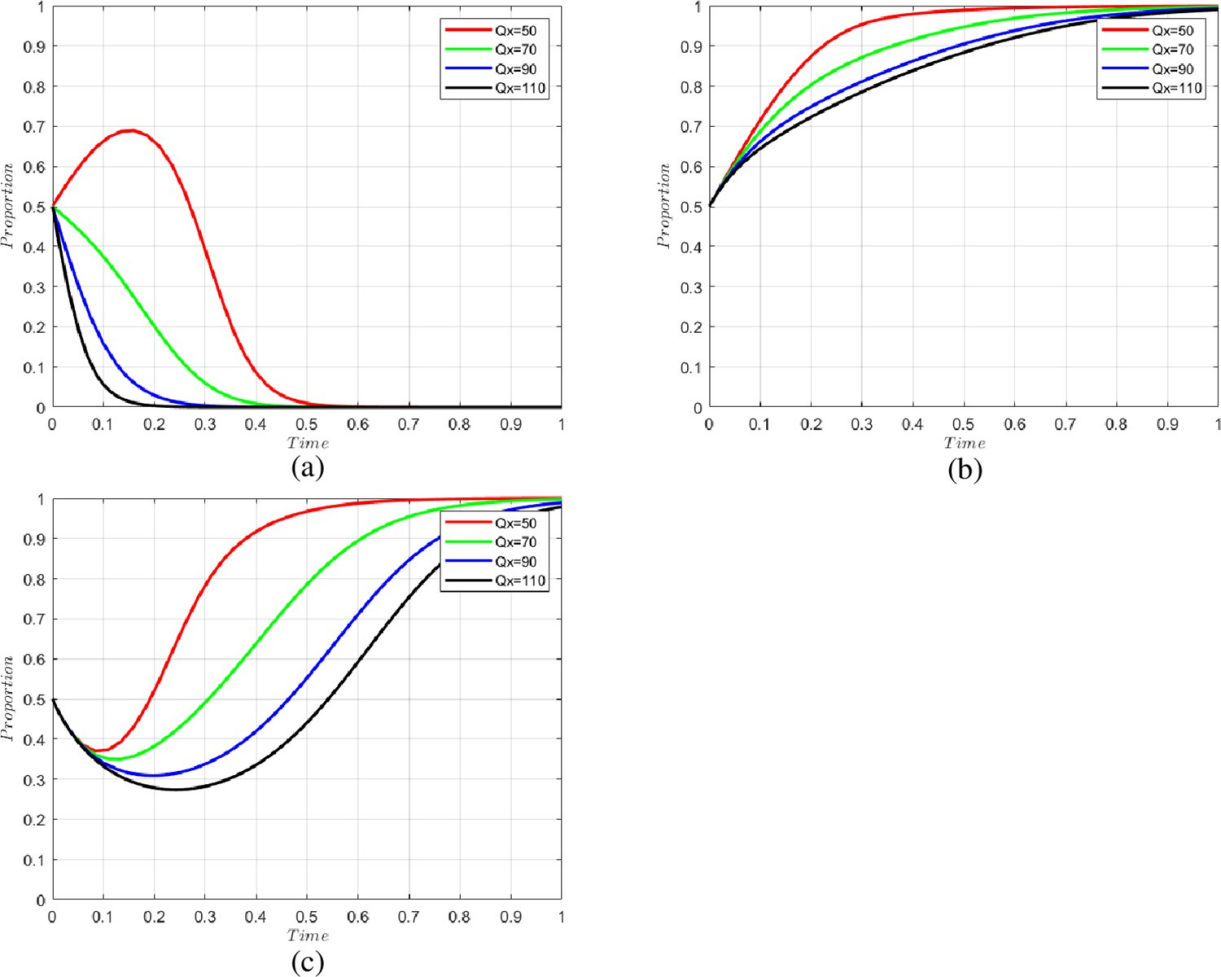
Changes in the willingness of parties to transport fresh produce in different quantities. (a) change in the willingness of n-level cold chain participants, (b) change in the willingness of consumers, and (c) change in the willingness of government.

We set the quantity of fresh agricultural products transported by cold chain as Qx = 50, 70, 90, 110; the change of willingness of each party is shown in Fig 4. As can be seen from the figure, the higher the quantity of fresh agricultural products transported in the cold chain logistics, the greater the willingness of the government to adopt blockchain technology and the n-level cold chain participants not to breach the contract. The larger the scale of cold chain logistics, the more that the government and n-level cold chain participants can reap benefits through the cold chain; thus, the government will choose to adopt blockchain technology, and n-level cold chain participants will choose not to default. The government should therefore actively promote the construction of the CCSCS and expand the scale of the cold chain.

#### 5.2.3 Impact of the chance of default of former cold chain participants

**Fig 5:**
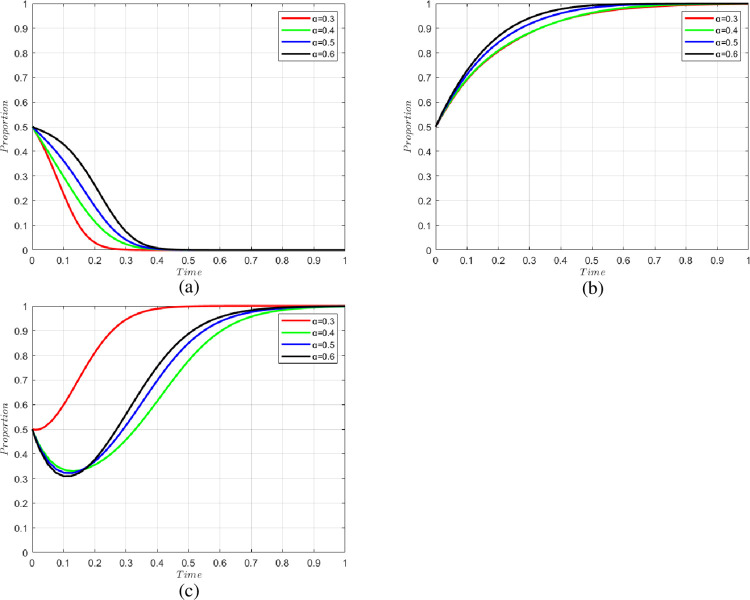
Changes in the willingness of the parties with different chances of default of the former cold chain participants. (a) change in the willingness of n-level cold chain participants, (b) change in the willingness of consumers, and (c) change in the willingness of government.

We set the default probability as α = 0.3, 0.4, 0.5, 0.6 for the former cold chain participants; the change of willingness of each party is shown in Fig 5. As can be seen from the figure, there are many nodes in the cold chain transportation, and the information for each node is not shared under the traditional cold chain system. Therefore, the behavior of a single cold chain participant will have significant externality, and the default of a particular cold chain participant will affect the willingness of other cold chain participants not to default. As can be seen from the figure, the higher the willingness to default of the pre-cold-chain participant, the lower the motivation of the n-level cold chain participant not to default. In addition, the higher the willingness of the former cold chain participants to default, the higher the necessity of the government to use blockchain technology.

#### 5.2.4 Impact of government on cold chain participants and consumer rewards

**Fig 6:**
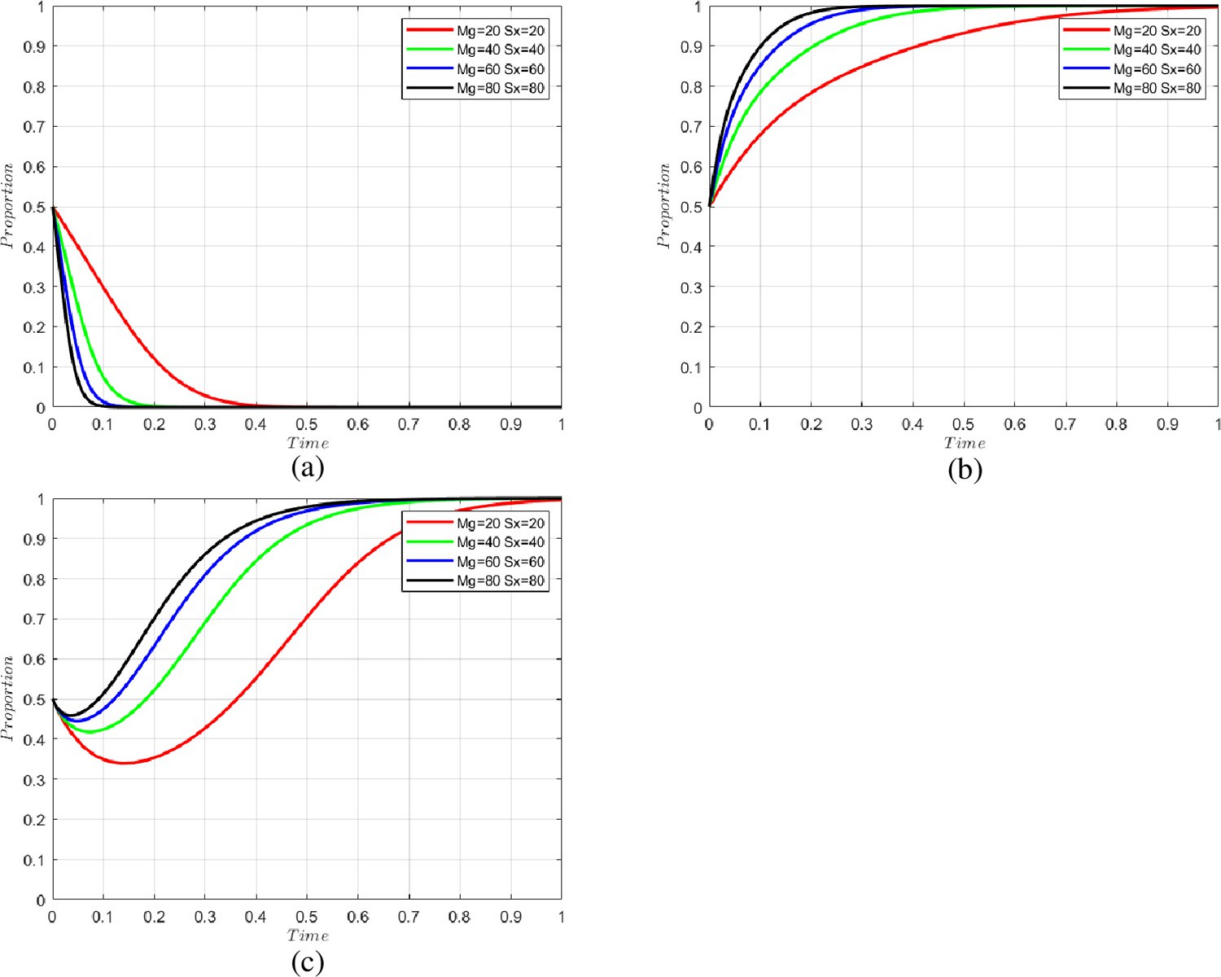
Changes in the willingness of parties with different government incentives. (a) change in the willingness of n-level cold chain participants, (b) change in the willingness of consumers, and (c) change in the willingness of the government.

We set the government rewards to cold chain participants and consumers as Mg = 20, 40, 60, 80 and Sx = 20, 40, 60, 80, respectively. The change in the willingness of each party is shown in Fig 6. From the figure, it can be seen that the increase in government incentives will have a positive impact on the non-violation behavior of cold chain participants and the willingness of consumers to report their behavior. In the early stage of industry construction, when the compliance awareness of cold chain participants is low and the food safety awareness of consumers is poor, the government’s reward policy will play a leading role. However, with the increase in government rewards, the effect will become less pronounced, and the impact of government rewards will become smaller over time. The government should set a reasonable incentive mechanism to achieve the maximum benefit.

## 6. Discussion

As a predominantly agricultural country, China ranks first in the world in producing fresh agricultural products such as perishable fruits and vegetables [16]. However, due to inadequate infrastructure and storage facilities and the lack of relevant knowledge and technology, the loss rate of fresh agricultural products in China has been high [59], with storage losses currently accounting for about 15–20% for vegetables and 10–15% for fruits. Improving traditional cold chain logistics and building a CCSCS requires introducing modern technology [60]. Building a CCSCS is a process involving coordinating multiple stakeholders [61]. The CCSCS requires a reliable information traceability system to effectively monitor the cold chain and protect the interests of various cold chain participants, including consumers. This means that the national government must coordinate the interests of multiple parties [62] to achieve the completion of the CCSCS.

Countries worldwide attach great importance to the construction of modern cold chain logistics. Canada was the first to build a complete set of complex and efficient integrated cold chain logistics systems, which organically combined air, land, rail, and waterways [63], with a highly developed cold chain logistics network for agricultural products formed by coordination among various carrier resources [64]. To lessen the environmental impact of storing and transporting food, a system of low-temperature controls must be implemented throughout the entire cold chain logistics operation. The network ensures that the loss of fruits and vegetables in Canada is controlled within 5% by modern means, and the logistics cost is less than 30%. The integration of cold chain resources has expanded the scale and transportation methods within the industry, effectively reducing transportation losses. This aligns with the sensitivity analysis in this paper regarding the quantity factor of fresh agricultural products transportation. Through strategic collaboration and resource allocation, the cold chain has optimized operations, improved coordination, and increased transportation capacity. As a result, losses in transportation have been significantly reduced, leading to a more efficient and sustainable cold chain system. The congruity between the sensitivity analysis and the discussions in this paper highlights the importance of integrating cold chain resources in addressing challenges related to the transportation of varying quantities of fresh agricultural products. The United States applies RFID, a GPS equipped with automatic temperature control [65], for the real-time temperature monitoring of pharmaceutical temperatures [66]. This system allows for maintaining the temperature of transported pharmaceuticals in the range of 2–8°C during the cold chain shipping process of vaccines, biological agents, and other medications. Additionally, the United States possesses the most cutting-edge "three-stage" refrigerated delivery trucks, which can simultaneously meet the temperature needs of three separate refrigerated medications [67]. For each cold chain vehicle operated by Britain’s C.R. England cold chain service transport company, an electronic data interchange, a satellite positioning system, a remote control platform, and other cutting-edge auxiliary technology are standard [68] and can monitor the whereabouts of each cold chain vehicle in real time, providing confirmation that the procedure meets standards for quality and safety. Japan is also a leader in barcode innovation [69] and temperature sensor technology, which can monitor the quality of pharmaceutical cold chain logistics services in real time. Similarly, Japan has implemented an in-vehicle mapping system to determine logistics distribution routes for pharmaceutical cold chain distribution vehicles, which greatly decreases the logistics of in-transit consumption time and improves the efficiency of pharmaceutical cold chain logistics distribution. The integration of digital technologies has ushered in significant advancements within the cold chain industry, leading to a more centralized, systematic, and transparent approach. This transformative shift has successfully mitigated the potential negative impact of individual participants on the entire system, thus fostering the overall development of the cold chain. The findings from the sensitivity analysis conducted in this paper regarding the default probability of previous cold chain participants provide further support for these observations. The analysis highlights the crucial importance of enhancing processes and promoting transparency throughout the cold chain, a goal that can be effectively realized through the integration of digital technologies. By leveraging tools such as real-time tracking, temperature monitoring, and data analytics, the cold chain ecosystem can proactively manage risks and optimize operational efficiency across the entire supply chain.

The necessity of using blockchain and other technologies in the cold chain has been proven in numerous studies. Smart contracts combining blockchain and IoT technologies applied in the blood cold chain (BCC) can ensure the quality of blood or blood products [70]. Using blockchain technology (BCT) in the food cold chain, is expected to provide traceability and sustainability for food products [71]. Wan [72] investigated a novel drug quality control technology for cold chain logistics based on port transportation to achieve strict control in the port logistics industry. The simulation results in this paper also show that the development of blockchain technology to reduce the costs associated with technology use can effectively facilitate the construction of CCSCSs. The combination of modern technology and traditional cold chain logistics makes all aspects of cold chain logistics more compact, builds an information traceability system, forms a CCSCS, and provides a development direction for the high-quality development of the cold chain industry. The simulation results of this paper also indicate that the government’s incentive policy plays a motivating role in encouraging positive behavior among all parties, aligning with existing research in the field. The government should set a reasonable reward and punishment mechanism [73], regulate the price of cold chain transportation products [74], promote the development of the cold chain, and support and manage the information sharing of companies, all of which can effectively prevent the phenomenon of cold chain disconnection [75].

## 7. Conclusions

An evolutionary game model involving n-level cold chain participants, consumers, and government is established in this paper using evolutionary game theory and prospect theory. We analyze the stability of the model and simulate the impact of different parameter changes on the system to reveal the effect of government blockchain technology usage on building a CCSCS. We draw the following three conclusions.

First, we determined three stability cases that will occur in the construction of the CCSCS as well as the reasons why for these cases through system stability analysis, of the early, middle, and late stages of CCSCS construction. Through this study, we conclude that factors such as government incentives and the cost of using blockchain technology in the process of CCSCS construction will affect the evolution of the system to a higher stage of development. Factors such as Ck, Qx, α, Mg, and Sx will influence the evolutionary steady state of the system.

Second, the research results show that there are strong negative externalities among the participants in the cold chain. At the same time, the scale of the cold chain will also have an impact on the system. The larger the scale of the cold chain, the greater the reduction in default of the cold chain participants. The cost of using block-chain technology will significantly affect the benefit of its adoption by the government, thus affecting the behavior of cold chain participants and consumers. In addition, the government’s incentive policy will also have a positive influence; a customized and reasonable incentive mechanism will have an essential impact in the early stage of cold chain supply chain construction.

Third, this paper combines prospect theory with evolutionary game theory. Through evolutionary game theory, we obtain the evolutionary stability strategy of the three parties of n-level cold chain participants, consumers, and government after continuous trial and error, imitation, and learning, while prospect theory adds the perceived differences in the consideration of the three parties with limited rationality; thus the stability strategy obtained can provide guidance for the construction of realistic cold chain supply chain system. In order to build a strong cold chain supply chain system, Chinese cold chain enterprises need to work to improve the overall awareness of non-breach of contract among all the nodes involved in the cold chain and build a positive environment to promote the construction of the cold chain supply chain system. Enterprises should also actively participate in the use of blockchain technology to jointly maintain a traceability system with transparent information.

### 7.1 Policy recommendations

Based on the conclusions obtained after the analysis of the system stability and sensitivity of typical parameters, we present the following policy recommendations.

First, publicity and education must be strenghtened. Promoting the Concept of Cold Chain Logistics, Raising Awareness about the Hazards of Cold Chain Disruptions, and Enhancing Public Understanding and Support for Initiatives to Advance Cold Chain Logistics. Cultivating good consumption habits and a healthy lifestyle, improve the ability of the public to identify unqualified frozen products, enhance the sense of responsibility of consumers to maintain the healthy development of the CCSCS, and build a good cold chain environment together through active reporting by consumers. At the same time to improve the cold chain enterprises and practitioners’ product quality and safety awareness, strict compliance with the cold chain logistics related laws and regulations and operating norms, building a solid cold chain product quality and safety line of defense. Publicize and promote several cold chain logistics enterprises should be encouraged to operate in good faith, with quality service in typical cases, to create a positive environment for industry development.

Second, the development of blockchain technology and associated technologies should be supported, as a boost in technological innovation will aid in the building of CCSCSs. Businesses involved in cold-chain logistics should be encouraged to ensure the timely delivery of data, informatization, and visualization of cold chain products and update, yard facilities, carrier equipment, and other infrastructure. Overall goals include lifting the bar on intelligent progress, pushing businesses toward cutting-edge IT and AI, and promoting the evolution and modernization of cold chain infrastructure, among others.

Third, a fair reward and punishment system should be implemented for the cold chain transportation regulatory system. The quality of cold chain transportation oversight should be improved by establishing a foolproof system for tracking shipments and increasing market surveillance. Use penalties for infractions, funding for the development of technology like blockchain, active compliance from cold chain members, and consumer reporting to ensure accountability are also important elements.

### 7.2 Limitations

In the process of constructing the three-way evolutionary game model of n-level cold chain participants, consumers, and government and its subsequent analysis, we realized some limitations of this paper, which will provide possible directions for our future research. First, this paper does not consider certain possible influencing factors regarding the construction of CCSCSs with blockchain technology, such as the psychological factors of cold chain participants influenced as by the negative externalities of other cold chain participants, as well as the additional costs required by cold chain participants. Second, this paper constructs the evolutionary game model based on three of n-level cold chain participants, consumers, and government, but does not consider the influence of more other stakeholders, such as fresh produce sellers on the construction of CCSCS. Moreover, the impact of consumers in the model is relatively smaller compared to the other two groups, and in the future, simplification of the model can be considered. Thirdly, this paper does not take into account the heterogeneity of individual behaviors of different subjects. In the future, we will address individual differences, such as the knowledge level and consumption demands of consumers and the professional ethics of cold chain participants.

## Supporting information

S1 Table Minimal data set. https://doi.org/10.6084/m9.figshare.24192573.v2.
(XLSX)

S1 File Replication of dynamic equations and eigenvalue computation procedures. https://doi.org/10.6084/m9.figshare.24197271.v4.
(DOCX)

S2 File The matlab program used to plot Fig 2. https://doi.org/10.6084/m9.figshare.24198243.v3.
(DOCX)

S3 File The matlab program used to plot Fig 3. https://doi.org/10.6084/m9.figshare.24198264.v3.
(DOCX)

S4 File The matlab program used to plot Fig 4. https://doi.org/10.6084/m9.figshare.24198273.v3.
(DOCX)

S5 File The matlab program used to plot Fig 5. https://doi.org/10.6084/m9.figshare.24198282.v3.
(DOCX)

S6 File The matlab program used to plot Fig 6. https://doi.org/10.6084/m9.figshare.24198288.v3.
(DOCX)
