# Supplementary material for: How to build a cold chain supply chain system for fresh agricultural products through blockchain technology—A study of tripartite evolutionary game theory based on prospect theory
Source: PLoS One. 2023 Nov 29;18(11):e0294520. doi: 10.1371/journal.pone.0294520 (PMC10686477; doi:10.1371/journal.pone.0294520)
Supplement: S1 File — https://doi.org/10.6084/m9.figshare.24197271.v4. (DOCX) [file pone.0294520.s002.docx]

function [out] = st(sy)

sy = sym(sy);

siz = prod(size(sy));

for i = 1:siz

in{i} = char(sy(i));

end

if siz == 1

in = char(in);

end

out = in;

clc;

syms x y z A B C n V(Sp) Pr Co Qu Qx V(Gb) Fc Mg Hl Hn V(Pe) Sx V(Mc) Ck Cf P Fx;

v11=y*z*(V(Sp)+Pr*Qx*(1-B)-Fc)+y*(1-z)*(V(Gb)+V(Sp)+Pr*Qx*(1-B)-Fc*C)+(1-y)*z*(V(Sp)+Pr*Qx*(1-B))+(1-y)*(1-z)*(V(Gb)+V(Sp)+Pr*Qx*(1-B));

v12=y*z*(V(Sp)+Pr*Qx+Mg-Co)+y*(1-z)*(V(Sp)+Pr*Qx+Mg-Co)+(1-y)*z*(V(Sp)+Pr*Qx-Co)+(1-y)*(1-z)*(V(Sp)+Pr*Qx-Co);

disp(' Total Expected Return for the First Subject: ')

v1=simplify(x*v11+(1-x)*v12)

disp(' Replicated dynamic equations for the first subject.')

fx=simplify(x*(v11-v1))

v21=x*z*(V(Pe)+Sx+V(Mc)-Hl-Hn)+x*(1-z)*(V(Pe)+Sx*C-Hl-Hn)+(1-x)*z*(V(Pe)+A*Sx+V(Mc)-Hl)+(1-x)*(1-z)*(V(Pe)+A*C*Sx-Hl);

v22=x*z*(V(Pe)+V(Mc)-Hl-Hn)+x*(1-z)*(V(Pe)-Hl-Hn)+(1-x)*z*(V(Pe)+V(Mc)-Hl)+(1-x)*(1-z)*(V(Pe)-Hl);

disp('Total Expected Returns for the Second Subject.')

v2=simplify(y*v21+(1-y)*v22)

disp(' Replicated dynamic equation for the second subject.')

fy=simplify(y*(v21-v2))

v31=x*y*(P-Ck+Fc-Sx+Fx)+x*(1-y)*(P-Ck-Fx)+(1-x)*y*(P-Ck+Fx-Mg)+(1-x)*(1-y)*(P-Ck);

v32=x*y*(P-Cf+Fc*C-Sx+Fx)+x*(1-y)*(P)+(1-x)*y*(P-Cf+Fx-Mg)+(1-x)*(1-y)*(P);

disp(' Total Expected Gain for the Third Subject.')

v3=simplify(z*v31+(1-z)*v32)

disp(' Replicated dynamic equation for the Third subject.')

fz=simplify(z*(v31-v3))

disp([' Jacobi Matrix']);

A = [diff(fx,x) diff(fx,y) diff(fx,z);

diff(fy,x) diff(fy,y) diff(fy,z);

diff(fz,x) diff(fz,y) diff(fz,z)]

equ=[fx==0,fy==0,fz==0];

answ=solve(equ,[x,y,z]);

disp([' Equalization point:']);A1=[answ.x, answ.y, answ.z];

%¶Ô¾ùºâµãÅÅÐò

A2=sortrows(A1,1)

disp([' Number of equalization points:']);length(A2)

xz=[];

for j=1:length(A2);

disp([' The ' num2str(j) '¸ first equilibrium point:']);

A2(j,:)

disp(['The' num2str(j) '¸Equilibrium points are substituted into the matrix after the Jacobi matrix:']);

x1=A2(j,1); y1=A2(j,2); z1=A2(j,3);

B=subs(A,[x y z],[x1 y1 z1])

disp(['The' num2str(j) '¸Eigenvalues of the matrix corresponding to the equilibrium point substitution:']);

[V,R] = eig(B);

B1=R(1,1)

B2=R(2,2)

B3=R(3,3)

xz=[xz;B1,B2,B3];

end

disp(['All eigenvalues corresponding to the equilibrium point']);xz

first=reshape(st([{'v11'} v11;{'v12'} v12;{'v1'} v1;{'fx'} fx]),4,2);

xlswrite(lujing,first,'The first subject expectation and replication dynamic equation')

second=reshape(st([{'v21'} v21;{'v22'} v22;{'v2'} v2;{'fy'} fy]),4,2);

xlswrite(lujing,second,' The Second subject expectation and replication dynamic equation ')

third=reshape(st([{'v31'} v31;{'v32'} v32;{'v3'} v3;{'fz'} fz]),4,2);

xlswrite(lujing,third,' The Third subject expectation and replication dynamic equation ')

yakebi=reshape(st(A),length(st(A))/3,3) ;

xlswrite(lujing,yakebi,' Jacobi Matrix ')

junhengdian=reshape(st(A2),length(st(A2))/3,3) ;

xlswrite(lujing,junhengdian,' balance point ')

tezhengzhi=reshape([st(xz)],length(st(xz))/3,3) ;

xlswrite(lujing,tezhengzhi,' Eigenvalues corresponding to equilibrium points ')

weifen=reshape(st([{'dwdt(1)'} fx;{'dwdt(2)'} fy;{'dwdt(3)'} fz]),3,2);

xlswrite(lujing,weifen,' differential equation (math.) ')
