# Supplementary material for: How to build a cold chain supply chain system for fresh agricultural products through blockchain technology—A study of tripartite evolutionary game theory based on prospect theory
Source: PLoS One. 2023 Nov 29;18(11):e0294520. doi: 10.1371/journal.pone.0294520 (PMC10686477; doi:10.1371/journal.pone.0294520)
Supplement: S2 File — https://doi.org/10.6084/m9.figshare.24198243.v3. (DOCX) [file pone.0294520.s003.docx]

%0,1,1

clc,clear;

figure(4);

A=0.2,B=0.2,C=0.3,n=4,Sp=80,Pr=3,Co=30,Qu=100,Qx=80,Gb=20,Fc=20,Mg=50,Hl=30,Hn=20,Pe=100,Sx=40,Mc=30,Ck=20,Cf=40,P=100,Fx=30;

[t,y]=ode45(@(t,y) lenlian1(t,y,A,B,C,n,Sp,Pr,Co,Qu,Qx,Gb,Fc,Mg,Hl,Hn,Pe,Sx,Mc,Ck,Cf,P,Fx),[0 1],[0.5 0.5 0.5]);

points=1:2:length(t);

plot(t,y(:,1),'r-','linewidth',2,'markersize',5,'markerfacecolor','r','markerindices',points);

hold on;

[t,y]=ode45(@(t,y) lenlian1(t,y,A,B,C,n,Sp,Pr,Co,Qu,Qx,Gb,Fc,Mg,Hl,Hn,Pe,Sx,Mc,Ck,Cf,P,Fx),[0 1],[0.5 0.5 0.5]);

points=1:2:length(t);

plot(t,y(:,2),'g-','linewidth',2,'markersize',5,'markerfacecolor','r','markerindices',points);

hold on;

[t,y]=ode45(@(t,y) lenlian1(t,y,A,B,C,n,Sp,Pr,Co,Qu,Qx,Gb,Fc,Mg,Hl,Hn,Pe,Sx,Mc,Ck,Cf,P,Fx),[0 1],[0.5 0.5 0.5]);

points=1:2:length(t);

plot(t,y(:,3),'b-','linewidth',2,'markersize',5,'markerfacecolor','r','markerindices',points);

hold on;

set(0,'defaultfigurecolor','w')

grid on

hold on

xlabel('$Time$','interpreter','latex','Rotation',0);

ylabel('$Proportion$','interpreter','latex');

set(gca,'XTick',[0:0.2:1],'YTick',[0:0.2:1])

axis([0 1 0 1]);

xlabel('$Time$','interpreter','latex','Rotation',0);

ylabel('$Proportion$','interpreter','latex');

set(gca,'XTick',[0:0.1:1],'YTick',[0:0.1:1])

axis([0 1 0 1]);

legend('=n-level cold chain participants','=consumers','=government');

title('','position',[0.5 -0.15]','FontWeight','bold');

%0,1,0

clc,clear;

figure(4);

A=0.2,B=0.2,C=0.3,n=4,Sp=80,Pr=3,Co=30,Qu=100,Qx=80,Gb=20,Fc=20,Mg=80,Hl=30,Hn=20,Pe=100,Sx=80,Mc=50,Ck=40,Cf=20,P=100,Fx=30;

[t,y]=ode45(@(t,y) lenlian1(t,y,A,B,C,n,Sp,Pr,Co,Qu,Qx,Gb,Fc,Mg,Hl,Hn,Pe,Sx,Mc,Ck,Cf,P,Fx),[0 1],[0.5 0.5 0.5]);

points=1:2:length(t);

plot(t,y(:,1),'r-','linewidth',2,'markersize',5,'markerfacecolor','r','markerindices',points);

hold on;

[t,y]=ode45(@(t,y) lenlian1(t,y,A,B,C,n,Sp,Pr,Co,Qu,Qx,Gb,Fc,Mg,Hl,Hn,Pe,Sx,Mc,Ck,Cf,P,Fx),[0 1],[0.5 0.5 0.5]);

points=1:2:length(t);

plot(t,y(:,2),'g-','linewidth',2,'markersize',5,'markerfacecolor','r','markerindices',points);

hold on;

[t,y]=ode45(@(t,y) lenlian1(t,y,A,B,C,n,Sp,Pr,Co,Qu,Qx,Gb,Fc,Mg,Hl,Hn,Pe,Sx,Mc,Ck,Cf,P,Fx),[0 1],[0.5 0.5 0.5]);

points=1:2:length(t);

plot(t,y(:,3),'b-','linewidth',2,'markersize',5,'markerfacecolor','r','markerindices',points);

hold on;

set(0,'defaultfigurecolor','w')

grid on

hold on

xlabel('$Time$','interpreter','latex','Rotation',0);

ylabel('$Proportion$','interpreter','latex');

set(gca,'XTick',[0:0.2:1],'YTick',[0:0.2:1])

axis([0 1 0 1]);

xlabel('$Time$','interpreter','latex','Rotation',0);

ylabel('$Proportion$','interpreter','latex');

set(gca,'XTick',[0:0.1:1],'YTick',[0:0.1:1])

axis([0 1 0 1]);

legend('=n-level cold chain participants','=consumers','=government');

title('','position',[0.5 -0.15]','FontWeight','bold');

%1,1,0

clc,clear;

figure(4);

A=0.2,B=0.2,C=0.3,n=4,Sp=80,Pr=3,Co=50,Qu=100,Qx=80,Gb=80,Fc=20,Mg=50,Hl=30,Hn=20,Pe=100,Sx=40,Mc=30,Ck=40,Cf=20,P=100,Fx=30;

[t,y]=ode45(@(t,y) lenlian1(t,y,A,B,C,n,Sp,Pr,Co,Qu,Qx,Gb,Fc,Mg,Hl,Hn,Pe,Sx,Mc,Ck,Cf,P,Fx),[0 1],[0.5 0.5 0.5]);

points=1:2:length(t);

plot(t,y(:,1),'r-','linewidth',2,'markersize',5,'markerfacecolor','r','markerindices',points);

hold on;

[t,y]=ode45(@(t,y) lenlian1(t,y,A,B,C,n,Sp,Pr,Co,Qu,Qx,Gb,Fc,Mg,Hl,Hn,Pe,Sx,Mc,Ck,Cf,P,Fx),[0 1],[0.5 0.5 0.5]);

points=1:2:length(t);

plot(t,y(:,2),'g-','linewidth',2,'markersize',5,'markerfacecolor','r','markerindices',points);

hold on;

[t,y]=ode45(@(t,y) lenlian1(t,y,A,B,C,n,Sp,Pr,Co,Qu,Qx,Gb,Fc,Mg,Hl,Hn,Pe,Sx,Mc,Ck,Cf,P,Fx),[0 1],[0.5 0.5 0.5]);

points=1:2:length(t);

plot(t,y(:,3),'b-','linewidth',2,'markersize',5,'markerfacecolor','r','markerindices',points);

hold on;

set(0,'defaultfigurecolor','w')

grid on

hold on

xlabel('$Time$','interpreter','latex','Rotation',0);

ylabel('$Proportion$','interpreter','latex');

set(gca,'XTick',[0:0.2:1],'YTick',[0:0.2:1])

axis([0 1 0 1]);

xlabel('$Time$','interpreter','latex','Rotation',0);

ylabel('$Proportion$','interpreter','latex');

set(gca,'XTick',[0:0.1:1],'YTick',[0:0.1:1])

axis([0 1 0 1]);

legend('=n-level cold chain participants','=consumers','=government');

title('','position',[0.5 -0.15]','FontWeight','bold');
