# Supplementary material for: How to build a cold chain supply chain system for fresh agricultural products through blockchain technology—A study of tripartite evolutionary game theory based on prospect theory
Source: PLoS One. 2023 Nov 29;18(11):e0294520. doi: 10.1371/journal.pone.0294520 (PMC10686477; doi:10.1371/journal.pone.0294520)
Supplement: S4 File — https://doi.org/10.6084/m9.figshare.24198273.v3. (DOCX) [file pone.0294520.s005.docx]

figure(9);

A=0.3,B=0.2,C=0.4,n=4,Sp=50,Pr=3,Co=45,Qu=100,Qx=50,Gb=20,Fc=20,Mg=20,Hl=13.5,Hn=15,Pe=100,Sx=20,Mc=30,Ck=30,Cf=40,P=100,Fx=18;

[t,y]=ode45(@(t,y) lenlian1(t,y,A,B,C,n,Sp,Pr,Co,Qu,Qx,Gb,Fc,Mg,Hl,Hn,Pe,Sx,Mc,Ck,Cf,P,Fx),[0 1],[0.5 0.5 0.5]);

points=1:2:length(t);plot(t,y(:,1),'r-','linewidth',2,'markersize',5,'markerfacecolor','r','markerindices',points);

hold on;

A=0.3,B=0.2,C=0.4,n=4,Sp=50,Pr=3,Co=45,Qu=100,Qx=70.5,Gb=20,Fc=20,Mg=20,Hl=13.5,Hn=15,Pe=100,Sx=20,Mc=30,Ck=30,Cf=40,P=100,Fx=18;

[t,y]=ode45(@(t,y) lenlian1(t,y,A,B,C,n,Sp,Pr,Co,Qu,Qx,Gb,Fc,Mg,Hl,Hn,Pe,Sx,Mc,Ck,Cf,P,Fx),[0 1],[0.5 0.5 0.5]);

points=1:2:length(t);plot(t,y(:,1),'g-','linewidth',2,'markersize',5,'markerfacecolor','r','markerindices',points);

hold on;

A=0.3,B=0.2,C=0.4,n=4,Sp=50,Pr=3,Co=45,Qu=100,Qx=90.5,Gb=20,Fc=20,Mg=20,Hl=13.5,Hn=15,Pe=100,Sx=20,Mc=30,Ck=30,Cf=40,P=100,Fx=18;

[t,y]=ode45(@(t,y) lenlian1(t,y,A,B,C,n,Sp,Pr,Co,Qu,Qx,Gb,Fc,Mg,Hl,Hn,Pe,Sx,Mc,Ck,Cf,P,Fx),[0 1],[0.5 0.5 0.5]);

points=1:2:length(t);plot(t,y(:,1),'b-','linewidth',2,'markersize',5,'markerfacecolor','r','markerindices',points);

hold on;

A=0.3,B=0.2,C=0.4,n=4,Sp=50,Pr=3,Co=45,Qu=100,Qx=110,Gb=20,Fc=20,Mg=20,Hl=13.5,Hn=15,Pe=100,Sx=20,Mc=30,Ck=30,Cf=40,P=100,Fx=18;

[t,y]=ode45(@(t,y) lenlian1(t,y,A,B,C,n,Sp,Pr,Co,Qu,Qx,Gb,Fc,Mg,Hl,Hn,Pe,Sx,Mc,Ck,Cf,P,Fx),[0 1],[0.5 0.5 0.5]);

points=1:2:length(t);plot(t,y(:,1),'k-','linewidth',2,'markersize',5,'markerfacecolor','r','markerindices',points);

hold on;

set(0,'defaultfigurecolor','w')

grid on

hold on

xlabel('$Time$','interpreter','latex','Rotation',0);

ylabel('$Proportion$','interpreter','latex');

set(gca,'XTick',[0:0.1:1],'YTick',[0:0.1:1])

axis([0 1 0 1]);

xlabel('$Time$','interpreter','latex','Rotation',0);

ylabel('$Proportion$','interpreter','latex');

set(gca,'XTick',[0:0.1:1],'YTick',[0:0.1:1])

axis([0 1 0 1]);

legend('Qx=50','Qx=70','Qx=90','Qx=110');

title('','position',[0.5 -0.15]','FontWeight','bold');

figure(10);

A=0.3,B=0.2,C=0.4,n=4,Sp=50,Pr=3,Co=45,Qu=100,Qx=50,Gb=20,Fc=20,Mg=20,Hl=13.5,Hn=15,Pe=100,Sx=20,Mc=30,Ck=30,Cf=40,P=100,Fx=18;

[t,y]=ode45(@(t,y) lenlian1(t,y,A,B,C,n,Sp,Pr,Co,Qu,Qx,Gb,Fc,Mg,Hl,Hn,Pe,Sx,Mc,Ck,Cf,P,Fx),[0 1],[0.5 0.5 0.5]);

points=1:2:length(t);plot(t,y(:,2),'r-','linewidth',2,'markersize',5,'markerfacecolor','r','markerindices',points);

hold on;

A=0.3,B=0.2,C=0.4,n=4,Sp=50,Pr=3,Co=45,Qu=100,Qx=70.5,Gb=20,Fc=20,Mg=20,Hl=13.5,Hn=15,Pe=100,Sx=20,Mc=30,Ck=30,Cf=40,P=100,Fx=18;

[t,y]=ode45(@(t,y) lenlian1(t,y,A,B,C,n,Sp,Pr,Co,Qu,Qx,Gb,Fc,Mg,Hl,Hn,Pe,Sx,Mc,Ck,Cf,P,Fx),[0 1],[0.5 0.5 0.5]);

points=1:2:length(t);plot(t,y(:,2),'g-','linewidth',2,'markersize',5,'markerfacecolor','r','markerindices',points);

hold on;

A=0.3,B=0.2,C=0.4,n=4,Sp=50,Pr=3,Co=45,Qu=100,Qx=90.5,Gb=20,Fc=20,Mg=20,Hl=13.5,Hn=15,Pe=100,Sx=20,Mc=30,Ck=30,Cf=40,P=100,Fx=18;

[t,y]=ode45(@(t,y) lenlian1(t,y,A,B,C,n,Sp,Pr,Co,Qu,Qx,Gb,Fc,Mg,Hl,Hn,Pe,Sx,Mc,Ck,Cf,P,Fx),[0 1],[0.5 0.5 0.5]);

points=1:2:length(t);plot(t,y(:,2),'b-','linewidth',2,'markersize',5,'markerfacecolor','r','markerindices',points);

hold on;

A=0.3,B=0.2,C=0.4,n=4,Sp=50,Pr=3,Co=45,Qu=100,Qx=110,Gb=20,Fc=20,Mg=20,Hl=13.5,Hn=15,Pe=100,Sx=20,Mc=30,Ck=30,Cf=40,P=100,Fx=18;

[t,y]=ode45(@(t,y) lenlian1(t,y,A,B,C,n,Sp,Pr,Co,Qu,Qx,Gb,Fc,Mg,Hl,Hn,Pe,Sx,Mc,Ck,Cf,P,Fx),[0 1],[0.5 0.5 0.5]);

points=1:2:length(t);plot(t,y(:,2),'k-','linewidth',2,'markersize',5,'markerfacecolor','r','markerindices',points);

hold on;

set(0,'defaultfigurecolor','w')

grid on

hold on

xlabel('$Time$','interpreter','latex','Rotation',0);

ylabel('$Proportion$','interpreter','latex');

set(gca,'XTick',[0:0.1:1],'YTick',[0:0.1:1])

axis([0 1 0 1]);

xlabel('$Time$','interpreter','latex','Rotation',0);

ylabel('$Proportion$','interpreter','latex');

set(gca,'XTick',[0:0.1:1],'YTick',[0:0.1:1])

axis([0 1 0 1]);

legend('Qx=50','Qx=70','Qx=90','Qx=110');

title('','position',[0.5 -0.15]','FontWeight','bold');

figure(11);

A=0.3,B=0.2,C=0.4,n=4,Sp=50,Pr=3,Co=45,Qu=100,Qx=50,Gb=20,Fc=20,Mg=20,Hl=13.5,Hn=15,Pe=100,Sx=20,Mc=30,Ck=30,Cf=40,P=100,Fx=18;

[t,y]=ode45(@(t,y) lenlian1(t,y,A,B,C,n,Sp,Pr,Co,Qu,Qx,Gb,Fc,Mg,Hl,Hn,Pe,Sx,Mc,Ck,Cf,P,Fx),[0 1],[0.5 0.5 0.5]);

points=1:2:length(t);plot(t,y(:,3),'r-','linewidth',2,'markersize',5,'markerfacecolor','r','markerindices',points);

hold on;

A=0.3,B=0.2,C=0.4,n=4,Sp=50,Pr=3,Co=45,Qu=100,Qx=70.5,Gb=20,Fc=20,Mg=20,Hl=13.5,Hn=15,Pe=100,Sx=20,Mc=30,Ck=30,Cf=40,P=100,Fx=18;

[t,y]=ode45(@(t,y) lenlian1(t,y,A,B,C,n,Sp,Pr,Co,Qu,Qx,Gb,Fc,Mg,Hl,Hn,Pe,Sx,Mc,Ck,Cf,P,Fx),[0 1],[0.5 0.5 0.5]);

points=1:2:length(t);plot(t,y(:,3),'g-','linewidth',2,'markersize',5,'markerfacecolor','r','markerindices',points);

hold on;

A=0.3,B=0.2,C=0.4,n=4,Sp=50,Pr=3,Co=45,Qu=100,Qx=90.5,Gb=20,Fc=20,Mg=20,Hl=13.5,Hn=15,Pe=100,Sx=20,Mc=30,Ck=30,Cf=40,P=100,Fx=18;

[t,y]=ode45(@(t,y) lenlian1(t,y,A,B,C,n,Sp,Pr,Co,Qu,Qx,Gb,Fc,Mg,Hl,Hn,Pe,Sx,Mc,Ck,Cf,P,Fx),[0 1],[0.5 0.5 0.5]);

points=1:2:length(t);plot(t,y(:,3),'b-','linewidth',2,'markersize',5,'markerfacecolor','r','markerindices',points);

hold on;

A=0.3,B=0.2,C=0.4,n=4,Sp=50,Pr=3,Co=45,Qu=100,Qx=110,Gb=20,Fc=20,Mg=20,Hl=13.5,Hn=15,Pe=100,Sx=20,Mc=30,Ck=30,Cf=40,P=100,Fx=18;

[t,y]=ode45(@(t,y) lenlian1(t,y,A,B,C,n,Sp,Pr,Co,Qu,Qx,Gb,Fc,Mg,Hl,Hn,Pe,Sx,Mc,Ck,Cf,P,Fx),[0 1],[0.5 0.5 0.5]);

points=1:2:length(t);plot(t,y(:,3),'k-','linewidth',2,'markersize',5,'markerfacecolor','r','markerindices',points);

hold on;

set(0,'defaultfigurecolor','w')

grid on

hold on

xlabel('$Time$','interpreter','latex','Rotation',0);

ylabel('$Proportion$','interpreter','latex');

set(gca,'XTick',[0:0.1:1],'YTick',[0:0.1:1])

axis([0 1 0 1]);

xlabel('$Time$','interpreter','latex','Rotation',0);

ylabel('$Proportion$','interpreter','latex');

set(gca,'XTick',[0:0.1:1],'YTick',[0:0.1:1])

axis([0 1 0 1]);

legend('Qx=50','Qx=70','Qx=90','Qx=110');

title('','position',[0.5 -0.15]','FontWeight','bold');
